# The impact of patient-reported factors of endoscopic screening experience on attendance at future examinations and distal colorectal cancer incidence

**DOI:** 10.1186/s12885-025-13771-3

**Published:** 2025-03-06

**Authors:** Sharon Power, Kate Wooldrage, Siwan Thomas-Gibson, Amanda J. Cross

**Affiliations:** 1 Cancer Screening and Prevention Research Group (CSPRG), Department of Surgery and Cancer, Imperial College London, London, UK; 2 Wolfson Unit for Endoscopy, St Mark’s Hospital, London, UK; 3 Department of Metabolism, Digestion and Reproduction, Imperial College London, London, UK

**Keywords:** Colonoscopy, Colorectal cancer, Flexible sigmoidoscopy, Patient experience, Patient satisfaction

## Abstract

**Background:**

Endoscopic examinations can reduce colorectal cancer (CRC) burden through early detection and removal of precancerous lesions; however, after initial endoscopy, some patients do not attend subsequent examinations.

**Aims:**

To investigate the impact of patient experience of endoscopic screening on attendance at future examinations and distal CRC incidence.

**Methods:**

In a cohort study including 40,141 participants who received flexible sigmoidoscopy (FS) screening in the UK FS Screening Trial, median follow-up was 16.8 years. We examined family history of CRC, bowel preparation quality, segment of bowel reached, and responses to patient-reported post-examination questionnaires. We estimated multivariable odds ratios (OR) for attendance at future examinations by logistic regression and hazard ratios (HR) for associations between patient experience at FS and distal CRC incidence.

**Results:**

Of those recommended a future endoscopy, 7.1% did not attend repeat FS, 3.4% did not attend colonoscopy, 18.3% did not attend surveillance, and 0.5% developed distal CRC. Symptoms of faintness/dizziness (OR = 5.10 95%CI 1.49–17.42) were associated with non-attendance at repeat FS. Non-attendance at surveillance was associated with whether participants felt they had made the right decision to take the tests; that taking the tests was tempting fate; that they needed the tests; or that they would rather have let nature take its course. A FS more painful than expected (HR = 0.57 95%CI 0.37–0.88) was inversely associated with distal CRC incidence.

**Conclusions:**

We identified aspects of patient experience at endoscopy that could be used to improve attendance at future endoscopic examinations, which in turn could reduce CRC incidence. Trial registration number: ISRCTN28352761. Trial registration date: April 2000.

**Supplementary Information:**

The online version contains supplementary material available at 10.1186/s12885-025-13771-3.

## Introduction

Colorectal cancer (CRC) is the fourth most common cancer with over 42,000 newly diagnosed cases in the UK annually [1]. Endoscopic examination can prevent CRC via the detection and removal of precursor lesions, as well as detect CRC earlier to improve outcomes [2]. There is strong evidence to support the effectiveness of endoscopic screening in reducing CRC incidence and mortality [3–6] but high patient adherence is essential to realise its full potential [7].

Previous experience at endoscopy can influence attendance at future examinations [8–10]. Individuals may be asked to return for a subsequent examination due to inadequate bowel preparation [11] or after removal of high-risk polyps [12]. Additionally, high-risk populations, such as those with Lynch syndrome, require regular surveillance examinations [13]. Previous research reported that approximately 25% of high-risk individuals delayed their examination by more than one year due to discomfort or embarrassment associated with endoscopy [14].

Individuals who feel satisfied with their endoscopic experience are more likely to return for repeat examinations [15]. Patient satisfaction with endoscopy has been associated with the endoscopist’s personal manner, the patient’s perception of the endoscopist’s technical skill, increased time with the clinician [16, 17], and pain management [17]. Higher levels of discomfort/pain are associated with decreased patient satisfaction [16]. Experiencing pain during endoscopic examination can affect an individual’s willingness to attend future endoscopies [8, 10, 14]. Among individuals invited to a five-year follow-up examination after a normal flexible sigmoidoscopy (FS), 65% of those who declined the follow-up cited pain and unpleasantness associated with FS as the reason for their decision [10].

A technically inadequate examination (endoscope inserted < 50 cm depth due to discomfort or visual inspection of < 90% of the mucosal surface due to bowel preparation, without detection of a polyp/mass) is a strong predictor of non-adherence to future examinations [9]. In addition, a shorter depth of insertion was associated with increased pain and less satisfaction [18]. Non-attendance or delaying endoscopic exams leaves any abnormalities in situ, which can increase CRC risk [19].

As repeat endoscopic examinations hold such importance in the prevention of CRC, it is key that we understand how patient experience impacts satisfaction and willingness to attend future examinations and the consequential impact on long-term outcomes. A positive first endoscopic experience could minimise the risk of non-attendance at future examinations, including at examinations required due to investigation of symptoms or participation in screening programmes, which could have implications on future CRC risk. Although FS without sedation is no longer offered as a primary screening tool in the UK, similar barriers to attending a FS, such as fear, pain, or discomfort, can be impediments to attendance at other endoscopic modalities [20] and, thus, could influence the success of screening programmes. Data from the UK Flexible Sigmoidoscopy Screening Trial (UKFSST) offers the opportunity to examine patient-reported experience of endoscopic screening and the association with attendance at future endoscopic examinations, including colonoscopy, and the impact on distal CRC incidence.

## Methods

### Study design and participants

The UKFSST recruited participants from 1994 to 1999 from general practices linked to 14 hospitals in the UK; details previously reported [4, 21–23]. Men and women aged 55–64 years were eligible unless they: were not able to provide informed consent; had a history of inflammatory bowel disease, adenomas, or CRC; had severe or terminal disease or life expectancy of less than 5 years; or had a sigmoidoscopy or colonoscopy within the previous three years. Eligible individuals were randomised to either the intervention arm (*n* = 57,237, invitation to once-only FS screening) or control arm (*n* = 113,195, no further contact).

All endoscopists used a standardised examination protocol in which they were asked to insert a 60 cm Olympus video-endoscope (CF-200 S) as far as possible without causing excessive pain or distress, usually to the sigmoid colon/descending colon junction, and to remove polyps less than 10 mm, leaving intact polyps less than 3 mm in the distal 4 cm of the rectum if thought to be hyperplastic [22]. Polyps ≥ 10 mm were to be removed at colonoscopy [22].

### Inclusion/exclusion criteria

**Fig. 1:**
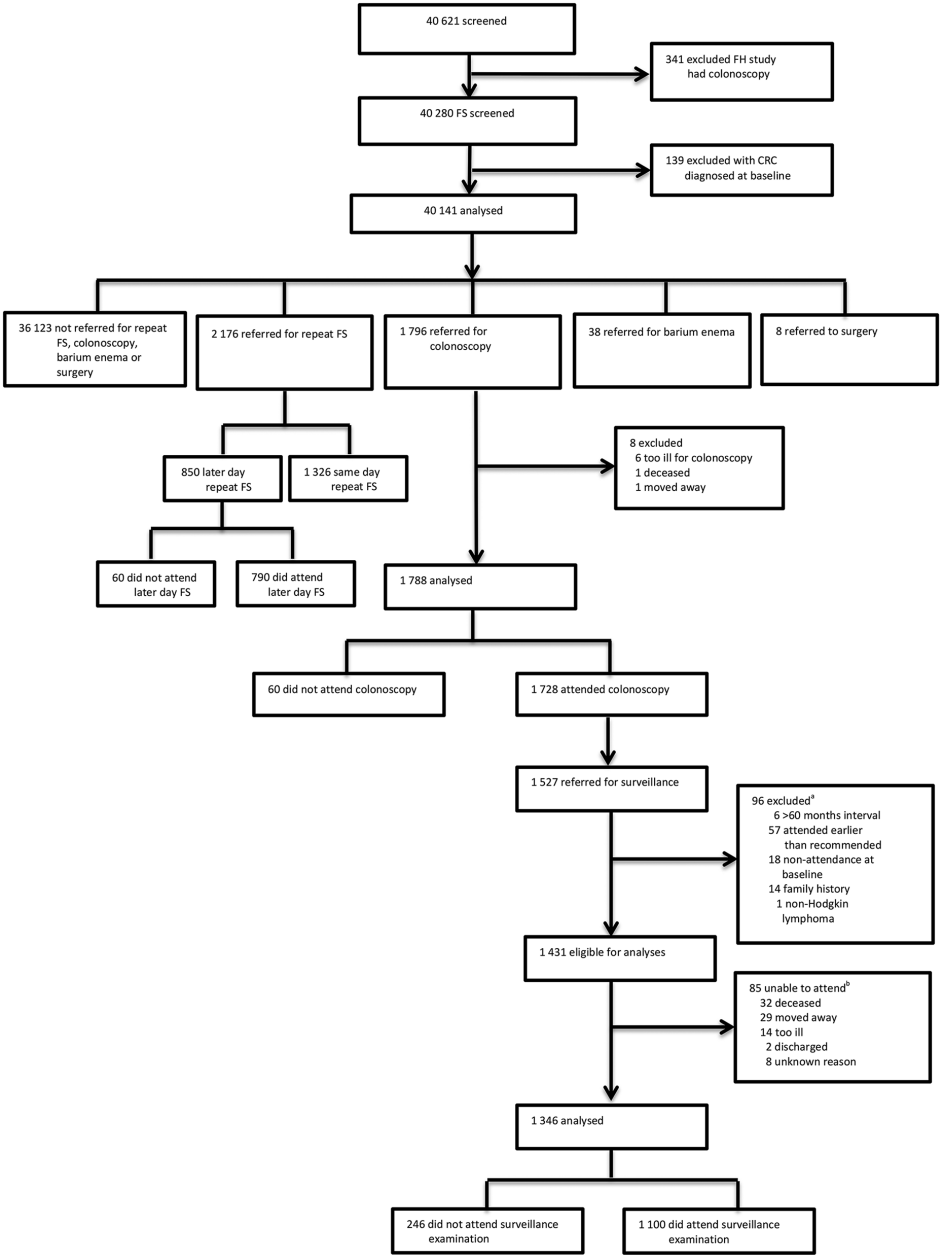
Study profile. Abbreviations: CRC = colorectal cancer. FH = family history. FS = flexible sigmoidoscopy. ^a^6 participants had a recommended surveillance interval > 60 months; 57 participants attended their surveillance > 6 months earlier than their recommended interval; 18 participants did not attend all referred baseline examinations; 14 were referred for surveillance due to a family history; 1 participant was diagnosed with non-Hodgkin’s lymphoma at baseline. ^b^32 participants had died before their examination; 29 had moved away from the area; 14 participants were too ill to attend their examination; 2 participants were discharged; 8 participants had no records to explain non-attendance

Among those in the intervention arm, 139 were excluded due to pre-randomisation death (*n* = 55) or CRC diagnosis (*n* = 77), duplicate study numbers (*n* = 6), or being outside of the age range (*n* = 1). Of the 57,098 eligible for analyses, 16,477 did not attend screening. Of the 40,621 attending, 341 participants were excluded due to participation in a sub-study where they received colonoscopy instead of FS at baseline and 139 participants were excluded due to CRC diagnosis at baseline. The remaining 40,141 participants were grouped according to their first referred procedure following the initial FS: eight were referred for surgery, 38 for barium enema (BE), 2,176 for repeat FS, 1,796 for colonoscopy, and 36,123 were not referred for repeat FS, BE, colonoscopy, or surgery (Fig. 1).

### Repeat FS examination

For those requiring a repeat FS (reasons for referral given in Supplementary Table 1), if time allowed and the participant agreed, this was conducted on the same day as the first FS (*n* = 1,326); when this was not possible, an appointment for a later day was scheduled (*n* = 850). The analysis of attendance at repeat FS only included participants whose first repeat examination was scheduled on a later day because those with a same day repeat were already present and in attendance. Only the first repeat FS was included in the analysis because an individual’s experience of FS could change over multiple examinations.

### Referred colonoscopy

Participants were referred for colonoscopy if they were suspected to be high-risk according to the trial protocol (those with ≥ 3 adenomas, a polyp ≥ 10 mm, an adenoma with villous/tubulovillous histology or high-grade dysplasia, malignant disease, or $\:\ge\:$20 hyperplastic polyps above the distal rectum) [21]. The analysis of attendance at referred colonoscopy only included participants with one prior FS as participant experience may alter over multiple examinations; 129 participants with multiple FS examinations prior to colonoscopy referral were excluded (61 same day repeat FS and 68 later day repeat FS). Of the 1,796 participants eligible for this analysis, eight were excluded (six were too ill to attend, one had died, and one had moved away), leaving 1,788 participants for analysis (Fig. 1).

### Surveillance colonoscopy

Participants confirmed to be high-risk after baseline colonoscopy were offered surveillance colonoscopy. Recall was usually at three years unless ≥ 5 adenomas or an adenoma ≥ 2 cm were found during baseline or baseline colonoscopy was technically unsatisfactory, then an additional colonoscopy was scheduled at 12 months. At the endoscopist’s discretion, an interval other than one or three years was sometimes recommended, often with no reason recorded. No further follow-up or surveillance was offered to low-risk participants [21].

Among 1,527 participants referred for surveillance colonoscopy, we excluded those who: had a recommended surveillance interval of > 60 months (*n* = 6), based on the longest recommended interval of 5 years in the 2002 UK adenoma surveillance guidelines [24]; attended surveillance more than six-months earlier than their recommended interval (*n* = 57); did not attend all referred baseline examinations (*n* = 18); were referred for surveillance due to a family history (*n* = 14); were diagnosed with non-Hodgkin’s lymphoma at baseline (*n* = 1); had died (*n* = 32) or moved away (*n* = 29); were too ill to attend their examination (*n* = 14); were discharged (*n* = 2); or had no records to explain non-attendance (*n* = 8). This left 1,346 participants for this analysis (Fig. 1). Recommended interval times were extended by 50% to allow for endoscopist/participant delays [25]. Participants who returned for their examination at an interval > 150% of the recommended interval were classified as non-attenders.

### CRC incidence

All eligible screened participants (*n* = 40,141) were included in these analyses.

### Exposures

All participants who attended FS screening were asked to complete a pre-examination questionnaire informing on their family history of CRC in first-degree relatives (yes, no) and a post-examination questionnaire, completed on the morning after FS, informing on their experience of the examination (Supplementary Table 2). Patient experience and satisfaction with colonoscopy were examined by responses provided on a post-colonoscopy questionnaire, sent to participants six months after their baseline colonoscopy (Supplementary Table 3). All baseline FS and colonoscopy examinations were scheduled to occur from 1994 to 2000 and first surveillance examinations from 1996 to 2005.

We also examined endoscopist-reported variables of bowel preparation quality (excellent, good, adequate, poor) (Supplementary Table 4) and segment of bowel reached (rectum, rectosigmoid, sigmoid colon, sigmoid-descending junction, descending colon, splenic flexure, transverse colon, hepatic flexure, ascending colon, caecum, terminal ileum) at FS and colonoscopy. In addition, we examined the endoscopist reported total procedure time for FS examinations, but only in association with other exposure variables. Age (years) at screening and sex (male, female) were also examined.

We investigated if age, sex, and the level of pain experienced by participants during FS were associated with the technical adequacy of the exam. We created an additional variable to identify if the first baseline FS examination was technically inadequate (yes, no), using a combination of the endoscopist reported variables of whether the exam was complete (to the sigmoid colon/descending colon junction; yes, no), the section of the bowel reached, and the quality of the bowel preparation. An inadequate examination was one satisfying any of the following: classed as incomplete; classed as unknown completeness but reached only the rectum, rectosigmoid, or sigmoid colon; or having poor bowel preparation. An adequate examination was one without poor bowel preparation that was either classed as complete or classed as unknown completeness and reached at least the sigmoid colon/descending colon junction.

### Outcome ascertainment

Primary outcomes were: (1) attendance at first repeat FS scheduled for a subsequent day (FS analysis); (2) attendance at first colonoscopy after one FS (colonoscopy analysis); (3) attendance at first surveillance after baseline colonoscopy (surveillance analysis); and (4) distal CRC incidence after the first baseline FS (CRC incidence analysis).

Distal CRCs were defined by the International Classification of Diseases 10th revision (ICD-10) codes as C18.7, C19, and C20 (rectum and sigmoid colon). CRC morphology was coded using the ICD for oncology 2nd edition and cancers included in the analysis were invasive adenocarcinomas and carcinomas not otherwise specified for cancers diagnosed on clinical grounds only [4]. For distal CRC incidence, the earliest distal CRC diagnosis per patient was used and the follow-up time for those participants receiving a proximal or unspecified site CRC diagnosis was not censored at their diagnosis.

### Statistical analysis

We examined associations between patient experience variables and attendance at future examinations and baseline characteristics and technically inadequate examinations using univariable and multivariable logistic regression to estimate odds ratios (OR) and 95% confidence intervals (CIs). To calculate hazard ratios (HR) and 95% CIs for distal CRC incidence, Cox models were used. Time-at-risk commenced from the first baseline FS and was censored at emigration, death, or the end of 2014 as the end of follow-up. To assess the assumption of proportionality, we used the Schoenfeld test; there was no evidence of any violations. Chi-squared (X^2^) tests were used to investigate associations between baseline characteristics and first referred procedure groups and between experiencing pain and symptoms at first FS examination and total procedure time.

Univariable analyses comprised of all eligible participants with complete data on each respective variable. A separate multivariable model was constructed for each exposure variable of interest, including all participants with data on the variable and with age and sex as covariates. Multivariable models for the FS, colonoscopy, and CRC incidence analyses also included potential confounders, identified using a one variable in, one variable out approach to determine which variables altered risk estimates by $\:\ge\:$10%. A missing category was included if data was missing for confounding variables. Examining collinearity revealed that test pain and expected pain were linearly related. Test pain was prioritised for inclusion in models as it was reported as part of the actual FS experience rather than as relative to that expected (Supplementary Tables 5–7).

For the surveillance analysis, several of the post-colonoscopy questions were interrelated; therefore, due to the risk of collinearity, each questionnaire variable was not assessed for inclusion as a covariate in multivariable models. Only the additional variables of family history, bowel preparation quality, and segment of the bowel reached were assessed for whether they had a confounding effect as these variables may influence patient experience and response.

STATA/IC V.13.1 (StataCorp LP, 2013; Stata Statistical Software: Release 13; Texas, USA) was used for statistical analyses. Two-sided p-values < 0.05 were deemed to be statistically significant. Ethical approval was acquired from the local research ethics review committees for each participating centre (Multicentre Research Ethics Committee reference number 03/01/22). The trial was registered (ISRCTN: 28352761) in April 2000. Written informed consent was given prior to FS examination for those in the intervention arm. Permission to obtain and process patient data was given by the Patient Information Advisory Group (PIAG 4–07(j)/2002). Access to the UKFSST full trial protocol is available online [26]. This study adheres to the STROBE guidelines.

## Results

### Flexible sigmoidoscopy

The median age of those referred for a subsequent day repeat FS (*n* = 850) was 60.7 years, 56.2% were males, and 13.5% had ≥ 1 first degree relative with CRC (Table 1). The median time from first FS to repeat FS was 31 days (IQR: 13–55). Compared to participants who were not referred for a follow-up examination (*n* = 36,123), those referred for a later day repeat FS were more likely to be male (56.2% vs. 49.2%), have had poor bowel preparation quality (68.5% vs. 1.7%), have had exams reaching only more distally (69.5% vs. 10.4% reaching only the rectum, rectosigmoid, or sigmoid colon), have experienced no pain (40.3% vs. 27.0%), have had less pain than expected (50.0% vs. 42.6%), or have had any symptoms of soiling (15.6% vs. 10.9%) (Supplementary Table 8).

**Table 1:** Non-attendance at referred FS examination among participants referred for a later day repeat FS by patient factors, bowel preparation quality, segment of the bowel reached, and questionnaire responses (*n* = 850)

	Referred for repeat FS *n* (%)^a^	Did not attend referred FS *n* (%)^a^	Univariable OR (95%CI)	*p*-value^b^	Multivariable OR (95%CI)^c^	*p*-value^b^
Total	850 (100)	60 (7.1)				
**Age**,** years**	**60.7 (58.1–63.0)**	**60.1 (57.9–63.2)**	0.98 (0.89–1.07)	0.59	-	-
**Sex**	**850 (100)**	**60 (7.1)**		0.20	-	-
Male	478 (56.2)	29 (6.1)	1		-	
Female	372 (43.8)	31 (8.3)	1.41 (0.83–2.38)		-	
**Family history of CRC** ^**d**^	**812 (95.5)**	**59 (7.3)**		0.09		0.18
No	702 (86.5)	55 (7.8)	1		1	
Yes	110 (13.5)	4 (3.6)	0.44 (0.16–1.25)		0.51 (0.18–1.47)	
**Bowel preparation quality at FS** ^**e**^	**839 (98.7)**	**60 (7.2)**		< 0.001		0.09
Excellent/good/adequate	264 (31.5)	7 (2.7)	1		1	
Poor	575 (68.5)	53 (9.2)	3.73 (1.67–8.31)		2.28 (0.84–6.16)	
**Segment reached at FS** ^**f**^	**812 (95.5)**	**58 (7.1)**		< 0.001		0.010
RM	91 (11.2)	10 (11.0)	1.84 (0.85-4.00)		1.61 (0.72–3.58)	
RS	132 (16.3)	20 (15.2)	2.66 (1.42-5.00)		2.66 (1.38–5.11)	
SC/SD	382 (47.0)	24 (6.3)	1		1	
DC/SF/TC/HF/AC/CM/TI	207 (25.5)	4 (1.9)	0.29 (0.10–0.86)		0.49 (0.14–1.67)	
**Post first FS questionnaire responses**						
**Test pain** ^**g**^	**715 (84.1)**	**45 (6.3)**		0.30		0.38
None	288 (40.3)	21 (7.3)	1		1	
Mild	331 (46.3)	16 (4.8)	0.65 (0.33–1.26)		0.63 (0.30–1.33)	
Quite a lot/severe	96 (13.4)	8 (8.3)	1.16 (0.49–2.70)		1.05 (0.36-3.00)	
**Expected pain** ^**h**^	**704 (82.8)**	**43 (6.1)**		0.40		0.29
Less painful	351 (50.0)	18 (5.1)	1		1	
About the same	251 (35.7)	16 (6.4)	1.26 (0.63–2.52)		1.18 (0.58–2.43)	
More painful	102 (14.5)	9 (8.8)	1.79 (0.78–4.12)		2.21 (0.85–5.78)	
**Abdominal pain or cramps** ^**i**^	**621 (74.1)**	**39 (6.3)**		0.39		0.71
None	431 (69.4)	24 (5.6)	1		1	
Mild	133 (21.4)	9 (6.8)	1.23 (0.56–2.72)		1.26 (0.48–3.33)	
Moderate/severe	57 (9.2)	6 (10.5)	2.00 (0.78–5.11)		1.71 (0.47–6.16)	
**Nausea or vomiting** ^**j**^	**563 (66.2)**	**34 (6.0)**		0.029		0.17
No symptoms	534 (94.9)	29 (5.4)	1		1	
Any symptoms	29 (5.2)	5 (17.2)	3.63 (1.29–10.20)		2.66 (0.69–10.25)	
**Faintness or dizziness** ^**k**^	**575 (67.6)**	**34 (5.9)**		0.049		0.013
No symptoms	519 (90.3)	27 (5.2)	1		1	
Any symptoms	56 (9.7)	7 (12.5)	2.60 (1.08–6.29)		5.10 (1.49–17.42)	
**Wind** ^**l**^	**652 (76.7)**	**39 (6.0)**		0.65		0.36
None	278 (42.6)	18 (6.5)	1		1	
Mild	245 (37.6)	12 (4.9)	0.74 (0.35–1.58)		0.58 (0.25–1.36)	
Moderate/severe	129 (19.8)	9 (7.0)	1.08 (0.47–2.48)		0.52 (0.17–1.57)	
**Bottom soreness** ^**m**^	**599 (70.5)**	**41 (6.8)**		0.26		0.43
None	406 (67.8)	23 (5.7)	1		1	
Mild	156 (26.0)	15 (9.6)	1.77 (0.90–3.49)		1.09 (0.47–2.52)	
Moderate/severe	37 (6.2)	3 (8.1)	1.47 (0.42–5.14)		0.40 (0.08–2.14)	
**Soiling** ^**n**^	**576 (67.8)**	**37 (6.4)**		0.32		0.65
No symptoms	486 (84.4)	29 (6.0)	1		1	
Any symptoms	90 (15.6)	8 (8.9)	1.54 (0.68–3.48)		0.78 (0.26–2.31)	
**Sleep disturbance** ^**o**^	**573 (67.4)**	**34 (5.9)**		0.69		0.028
No symptoms	511 (89.2)	31 (6.1)	1		1	
Any symptoms	62 (10.8)	3 (4.8)	0.79 (0.23–2.65)		0.19 (0.04–0.96)	

Abbreviations: AC = ascending colon. CI = confidence interval. CM = caecum. CRC = colorectal cancer. DC = descending colon. FS = flexible sigmoidoscopy. HF = hepatic flexure. OR = Odds ratio. RM = rectum. RS = recto sigmoid. SC = sigmoid colon. SD = sigmoid descending. SF = splenic flexure. TC = transverse colon. TI = terminal ileum

^a^All n and percentage except the entry for age, which is median and interquartile range

^b^Calculated with the likelihood ratio test

^c^A separate multivariable model was constructed for each specified variable, which included age, sex, the specified variable, and any additional variables classified as having a confounding effect, identified using a one variable in, one variable out approach to determine which variables altered the risk estimates by $\:\ge\:$10%. For confounding variables, a missing category was created for those with missing values, although it was necessary to exclude further participants from some multivariable models due to a lack of events in the missing category

^d^Family history multivariable model also includes segment of the bowel reached

^e^Bowel preparation quality multivariable model also includes test pain, faintness or dizziness, and segment of the bowel reached

^f^Segment of the bowel reached multivariable model also includes expected pain and bowel preparation quality (participants missing data on bowel preparation quality excluded, *n* = 804 included)

^g^Test pain multivariable model also includes abdominal pain or cramps, nausea or vomiting, faintness or dizziness, bottom soreness, bowel preparation quality, and segment of the bowel reached (participants missing data on bowel preparation quality excluded, *n* = 706 included)

^h^Expected pain multivariable model also includes abdominal pain or cramps, faintness or dizziness, bowel preparation quality, and segment of the bowel reached (participants missing data on bowel preparation quality excluded, *n* = 696 included)

^i^Abdominal pain or cramps multivariable model also includes test pain, nausea or vomiting, faintness or dizziness, bottom soreness, sleep disturbance, and bowel preparation quality (participants missing data on test pain or bowel preparation quality excluded, *n* = 607 included)

^j^Nausea or vomiting multivariable model also includes expected pain, abdominal pain or cramps, faintness or dizziness, sleep disturbance, and segment reached (participants missing data on abdominal pain or cramps or faintness or dizziness excluded, *n* = 551 included)

^k^Faintness or dizziness multivariable model also includes expected pain, abdominal pain or cramps, nausea or vomiting, sleep disturbance, bowel preparation quality, and segment of the bowel reached (participants missing data on abdominal pain or cramps, nausea or vomiting, or bowel preparation quality excluded, *n* = 544 included)

^l^Wind multivariable model also includes expected pain, abdominal pain or cramps, nausea or vomiting, faintness or dizziness, and bottom soreness

^m^Bottom soreness multivariable model also includes expected pain, abdominal pain or cramps, nausea or vomiting, faintness or dizziness, soiling, and segment of the bowel reached

^n^Soiling multivariable model also includes abdominal pain or cramps, nausea or vomiting, faintness or dizziness, and bottom soreness (participants missing data on bottom soreness excluded, *n* = 564 included)

^o^Sleep disturbance multivariable model also includes test pain, abdominal pain or cramps, nausea or vomiting, faintness or dizziness, bottom soreness, and bowel preparation quality (participants missing data on test pain, abdominal pain or cramps, bottom soreness, or bowel preparation excluded, *n* = 542 included)

There were 60 (7.1%) participants who did not attend their subsequent day FS (Table 1). Compared to reaching the sigmoid colon/sigmoid-descending junction, reaching only the rectosigmoid was associated with higher odds of non-attendance (multivariable: OR 2.66, 95%CI 1.38–5.11). Participants reporting any symptoms of faintness/dizziness (multivariable: OR 5.10, 95%CI 1.49–17.42) had increased odds of non-attendance compared to those not reporting these symptoms. Although there was an inverse association between symptoms of sleep disturbance and attendance at subsequent FS, only three participants reporting this symptom did not attend subsequent FS. Family history of CRC, bowel preparation quality, test pain, expected pain, abdominal pain/cramps, nausea/vomiting, wind, bottom soreness, and soiling were not associated with non-attendance at repeat FS in multivariable models (Table 1).

As reaching only the rectosigmoid colon was associated with non-attendance at a repeat FS and a technically inadequate examination has been associated with female sex, increasing age, and discomfort [27], we investigated whether these baseline characteristics were associated with technically inadequate FS examinations in our dataset. We found increasing age (per year: OR 1.03, 95%CI 1.02–1.04), female sex (females vs. males: OR 1.95, 95%CI 1.84–2.06), and increasing amounts of reported test pain (severe vs. no test pain: multivariable OR 4.04, 95%CI 3.50–4.66) were associated with a higher risk of experiencing a technically inadequate examination (Supplementary Table 9).

### Colonoscopy

The median age of those referred for colonoscopy after one FS (*n* = 1,788) was 60.8 years and 68.2% were males (Table 2). The median time from FS to colonoscopy was 48 days (IQR: 25–78). Compared to non-referred participants (*n* = 36,123), those referred for colonoscopy were more likely to be male (68.2% vs. 49.2%), to have a family history of CRC (14.5% vs. 11.4%), to have had poor bowel preparation quality (4.9% vs. 1.7%) or exams reaching only more distally (14.4% vs. 10.4% reaching only the rectum, rectosigmoid, or sigmoid colon), or to have experienced moderate/severe wind (22.5% vs. 20.0%), mild or moderate/severe bottom soreness (25.2% vs. 23.3%; 7.7% vs. 6.1%, respectively), or any symptoms of sleep disturbance (12.9% vs. 9.5%) (Supplementary Table 8). There were 60 (3.4%) participants who did not attend colonoscopy. There were no significant associations between patient-reported factors and non-attendance at colonoscopy (Table 2).

**Table 2:** Non-attendance at referred colonoscopy among participants referred for colonoscopy by patient factors, bowel preparation quality, segment of the bowel reached, and questionnaire responses (*n* = 1,788)

	Referred for colonoscopy *n* (%)^a^	Did not attend referred colonoscopy *n* (%)^a^	Univariable OR (95%CI)	*p*-value^b^	Multivariable OR (95%CI) ^c^	*p*-value^b^
Total	1,788 (100)	60 (3.4)				
**Age**,** years**	**60.8 (58.2–63.3)**	**61.5 (58.3–63.7)**	1.05 (0.96–1.15)	0.26	-	-
**Sex**	**1**,**788 (100)**	**60 (3.4)**		0.24	-	-
Male	1,219 (68.2)	45 (3.7)	1		-	
Female	569 (31.8)	15 (2.6)	0.71 (0.39–1.28)			
**Family history of CRC**	**1**,**660 (92.8)**	**55 (3.3)**		0.70		0.68
No	1,419 (85.5)	48 (3.4)	1		1	
Yes	241 (14.5)	7 (2.9)	0.85 (0 0.38-1.91)		0.85 (0.38–1.90)	
**Bowel preparation quality at FS**	**1**,**687 (94.4)**	**56 (3.3)**		> 0.99		0.95
Excellent/good	1,265 (75.0)	42 (3.3)	1		1	
Adequate/poor	422 (25.0)	14 (3.3)	1.00 (0.54–1.85)		0.98 (0.53–1.81)	
**Segment reached at FS**	**1**,**752 (98.0)**	**60 (3.4)**		0.45		0.54
RM/RS/SC/SD	543 (31.0)	16 (2.9)	1		1	
DC/SF/TC/HF/AC/CM/TI	1,209 (69.0)	44 (3.6)	1.24 (0.70–2.22)		1.20 (0.66–2.16)	
**Post first FS questionnaire responses**						
**Test pain**	**1**,**731 (96.8)**	**59 (3.4)**		0.82		0.77
None	502 (29.0)	16 (3.2)	1		1	
Mild	902 (52.1)	30 (3.3)	1.05 (0.56–1.94)		1.08 (0.58-2.00)	
Quite a lot/severe	327 (18.9)	13 (4.0)	1.26 (0.60–2.65)		1.31 (0.62–2.78)	
**Expected pain** ^**d**^	**1**,**725 (96.5)**	**57 (3.3)**		0.23		0.20
Less painful	741 (43.0)	29 (3.9)	1		1	
About the same	636 (36.9)	15 (2.4)	0.59 (0.32–1.12)		0.57 (0.30–1.08)	
More painful	348 (20.2)	13 (3.7)	0.95 (0.49–1.86)		0.89 (0.44–1.82)	
**Abdominal pain or cramps** ^**e**^	**1**,**582 (88.5)**	**53 (3.4)**		0.58		0.57
None	1,063 (67.2)	35 (3.3)	1		1	
Mild	374 (23.6)	11 (2.9)	0.89 (0.45–1.77)		0.67 (0.31–1.47)	
Moderate/severe	145 (9.2)	7 (4.8)	1.49 (0.65–3.42)		0.95 (0.35–2.53)	
**Nausea or vomiting** ^**f**^	**1**,**469 (82.2)**	**46 (3.1)**		0.30		0.29
No symptoms	1,394 (94.9)	42 (3.0)	1		1	
Symptoms	75 (5.1)	4 (5.3)	1.81 (0.63–5.20)		2.00 (0.60–6.67)	
**Faintness or dizziness** ^**g**^	**1**,**485 (83.1)**	**47 (3.2)**		0.75		0.72
No symptoms	1,317 (88.7)	41 (3.1)	1		1	
Symptoms	168 (11.3)	6 (3.6)	1.15 (0.48–2.76)		0.83 (0.29–2.34)	
**Wind** ^**h**^	**1**,**655 (92.6)**	**55 (3.3)**		0.87		0.93
None	621 (37.5)	20 (3.2)	1		1	
Mild	661 (39.9)	21 (3.2)	0.99 (0.53–1.84)		0.90 (0.47–1.70)	
Moderate/severe	373 (22.5)	14 (3.8)	1.17 (0.58–2.35)		1.00 (0.48–2.09)	
**Bottom soreness** ^**i**^	**1**,**547 (86.5)**	**49 (3.2)**		0.30		0.20
None	1,038 (67.1)	28 (2.7)	1		1	
Mild	390 (25.2)	17 (4.4)	1.64 (0.89–3.04)		1.78 (0.95–3.30)	
Moderate/severe	119 (7.7)	4 (3.4)	1.25 (0.43–3.64)		1.46 (0.48–4.42)	
**Soiling**	**1**,**486 (83.1)**	**45 (3.0)**		0.81		0.77
No symptoms	1,305 (87.8)	39 (2.8)	1		1	
Any symptoms	181 (12.2)	6 (3.3)	1.11 (0.46–2.67)		1.14 (0.48–2.75)	
**Sleep disturbance**	**1**,**495 (83.6)**	**44 (2.9)**		0.75		0.79
No symptoms	1,302 (87.1)	39 (3.0)	1		1	
Any symptoms	193 (12.9)	5 (2.6)	0.86 (0.34–2.21)		0.88 (0.34–2.28)	

Abbreviations: AC = ascending colon. CI = confidence interval. CM = caecum. CRC = colorectal cancer. DC = descending colon. FS = flexible sigmoidoscopy. HF = hepatic flexure. OR = Odds ratio. RM = rectum. RS = recto sigmoid. SC = sigmoid colon. SD = sigmoid descending. SF = splenic flexure. TC = transverse colon. TI = terminal ileum

^a^All n and percentage except the entry for age, which is median and interquartile range

^b^Calculated with the likelihood ratio test

^c^A separate multivariable model was constructed for each specified variable, which included age, sex, the specified variable, and any additional variables classified as having a confounding effect, identified using a one variable in, one variable out approach to determine which variables altered the risk estimates by $\:\ge\:$10%. For confounding variables, a missing category was created for those with missing values

^d^Expected pain multivariable model also includes abdominal pain or cramps

^e^Abdominal pain or cramps multivariable model also includes nausea or vomiting and bottom soreness

^f^Nausea or vomiting multivariable model also includes faintness or dizziness

^g^Faintness or dizziness multivariable model also includes nausea or vomiting

^h^Wind multivariable model also includes bottom soreness

^i^Bottom soreness multivariable model also includes expected pain

### Surveillance colonoscopy

The median age of those referred for surveillance colonoscopy (*n* = 1,346) was 60.7 years and 69.4% were males (Table 3). There were 246 (18.3%) who did not attend surveillance; attendance did not differ by age, sex, or family history of CRC.

**Table 3:** Non-attendance at referred surveillance examination among participants referred for surveillance by patient factors, bowel preparation quality, segment of the bowel reached, and questionnaire responses (*n* = 1,346)

	Referred for surveillance *n* (%)^a^	Did not attend referred surveillance examination *n* (%)^a^	Univariable OR (95%CI)	*p*-value^b^	Multivariable OR (95%CI)^c^	*p*-value^b^
Total	1,346 (100)	246 (18.3)				
**Age**,** years**	**60.7 (58.2–63.3)**	**60.9 (58.7–63.3)**	1.03 (0.98–1.08)	0.19	-	-
**Sex**	**1**,**346 (100)**	**246 (18.3)**		0.91		-
Male	934 (69.4)	170 (18.2)	1		-	
Female	412 (30.6)	76 (18.4)	1.02 (0.75–1.37)		-	
**Family history of CRC**	**1**,**258 (93.5)**	**227 (18.0)**		0.84		0.86
No	1,086 (86.3)	195 (18.0)	1		1	
Yes	172 (13.7)	32 (18.6)	1.04 (0.69–1.58)		1.04 (0.69–1.57)	
**Bowel preparation quality at baseline colonoscopy**	**1**,**267 (94.1)**	**227 (17.9)**		0.54		0.53
Excellent	305 (24.1)	47 (15.4)	1		1	
Good	627 (49.5)	118 (18.8)	1.27 (0.88–1.84)		1.28 (0.88–1.85)	
Adequate	279 (22.0)	50 (17.9)	1.20 (0.77–1.85)		1.20 (0.78–1.86)	
Poor	56 (4.4)	12 (21.4)	1.50 (0.74–3.04)		1.51 (0.74–3.07)	
**Segment reached at baseline colonoscopy**	**1**,**256 (93.1)**	**237 (18.9)**		0.002		0.002
RM/RS/SC/DC/SF/TC/HF/AC	106 (8.4)	33 (31.1)	2.10 (1.35–3.25)		2.06 (1.33–3.20)	
CM/TI	1,150 (91.6)	204 (17.7)	1		1	
**Post baseline colonoscopy questionnaire**						
**How satisfied were you with the information you were given before your colonoscopy?**	**1**,**062 (78.9)**	**194 (18.3)**		0.09		0.09
Very satisfied	616 (58.1)	102 (16.6)	1		1	
Satisfied	413 (38.9)	82 (19.9)	1.25 (0.90–1.72)		1.25 (0.91–1.73)	
Dissatisfied/very dissatisfied	33 (3.1)	10 (30.3)	2.19 (1.01–4.74)		2.25 (1.04–4.88)	
**How satisfied were you with the way the results of the colonoscopy were explained to you?** ^**d**^	**1**,**056 (78.5)**	**193 (18.3)**		0.19		0.18
Very satisfied	608 (57.6)	105 (17.3)	1		1	
Satisfied	397 (37.6)	74 (18.6)	1.10 (0.79–1.52)		1.08 (0.77–1.50)	
Dissatisfied	33 (3.1)	7 (21.2)	1.29 (0.55–3.05)		1.11 (0.46–2.71)	
Very dissatisfied	18 (1.7)	7 (38.9)	3.05 (1.15–8.05)		3.19 (1.21–8.45)	
**The tests gave me peace of mind?** ^**e**^	**1**,**059 (78.7)**	**195 (18.4)**		0.08		0.11
Strongly disagree/disagree	15 (1.4)	5 (33.3)	2.67 (0.89-8.00)		2.23 (0.72–6.87)	
Not sure	51 (4.8)	9 (17.6)	1.14 (0.54–2.43)		1.00 (0.46–2.16)	
Agree	442 (41.7)	94 (21.3)	1.44 (1.04–1.99)		1.43 (1.03–1.98)	
Strongly agree	551 (52.0)	87 (15.8)	1		1	
**Having the tests reduced my chance of getting bowel cancer?**	**1**,**039 (77.2)**	**190 (18.3)**		0.15		0.16
Strongly disagree	20 (1.9)	7 (35.0)	2.56 (0.99–6.64)		2.55 (0.98–6.60)	
Disagree	27 (2.6)	6 (22.2)	1.36 (0.53–3.48)		1.36 (0.53–3.49)	
Not sure	152 (14.6)	35 (23.0)	1.42 (0.91–2.24)		1.42 (0.90–2.24)	
Agree	408 (39.3)	67 (16.4)	0.94 (0.65–1.34)		0.94 (0.65–1.35)	
Strongly agree	432 (41.6)	75 (17.4)	1		1	
**I made the right decisions to take the tests?**	**1**,**061 (78.8)**	**195 (18.4)**		0.002		0.002
Strongly disagree/disagree	10 (0.9)	5 (50.0)	5.29 (1.51–18.57)		5.28 (1.50-18.56)	
Not sure	17 (1.6)	7 (41.2)	3.70 (1.38–9.93)		3.74 (1.39–10.04)	
Agree	336 (31.7)	72 (21.4)	1.44 (1.04–2.01)		1.44 (1.04–2.01)	
Strongly agree	698 (65.8)	111 (15.9)	1		1	
**The tests reassured me?** ^**f**^	**1**,**047 (77.8)**	**191 (18.2)**		0.65		0.60
Strongly disagree/disagree	15 (1.4)	4 (26.7)	1.78 (0.55–5.72)		1.65 (0.50–5.41)	
Not sure	58 (5.5)	11 (19.0)	1.15 (0.57–2.30)		0.98 (0.48–2.01)	
Agree	449 (42.9)	87 (19.4)	1.18 (0.85–1.63)		1.22 (0.87–1.69)	
Strongly agree	525 (50.1)	89 (17.0)	1		1	
**Having the tests made me feel that I was doing something positive about my health?** ^**g**^	**1**,**058 (78.6)**	**192 (18.1)**		0.22		0.22
Strongly disagree/disagree	23 (2.2)	3 (13.0)	0.75 (0.22–2.57)		0.66 (0.19–2.29)	
Not sure	11 (1.0)	4 (36.4)	2.86 (0.82–9.94)		2.66 (0.74–9.59)	
Agree	424 (40.1)	85 (20.0)	1.25 (0.91–1.73)		1.26 (0.91–1.74)	
Strongly agree	600 (56.7)	100 (16.7)	1		1	
**A screening test for bowel cancer is important?**	**1**,**057 (78.5)**	**191 (18.1)**		0.27		0.26
Strongly disagree/disagree	19 (1.8)	3 (15.8)	0.94 (0.27–3.27)		0.95 (0.27–3.32)	
Not sure	8 (0.8)	2 (25.0)	1.67 (0.33–8.37)		1.66 (0.33–8.31)	
Agree	279 (26.4)	61 (21.9)	1.40 (0.99–1.97)		1.40 (1.00-1.98)	
Strongly agree	751 (71.1)	125 (16.6)	1		1	
**Do you think that your experience of having the Flexi-scope test and colonoscopy has made you feel more relaxed?**	**1**,**042 (77.4)**	**192 (18.4)**		0.56		0.57
Not at all	92 (8.8)	14 (15.2)	0.81 (0.43–1.50)		0.82 (0.44–1.53)	
A little bit	129 (12.4)	29 (22.5)	1.31 (0.81–2.11)		1.32 (0.81–2.13)	
Quite a bit	392 (37.6)	71 (18.1)	1.00 (0.70–1.42)		1.00 (0.70–1.43)	
A great deal	429 (41.2)	78 (18.2)	1		1	
**Do you think that your experience of having the Flexi-scope test and colonoscopy has led to improved relationships with friends or relations?**	**991 (73.6)**	**186 (18.8)**		0.98		0.98
Not at all	634 (64.0)	119 (18.8)	1.01 (0.59–1.76)		1.02 (0.59–1.77)	
A little bit	136 (13.7)	27 (19.9)	1.09 (0.56–2.11)		1.09 (0.56–2.13)	
Quite a bit	124 (12.5)	22 (17.7)	0.95 (0.48–1.88)		0.95 (0.48–1.89)	
A great deal	97 (9.8)	18 (18.6)	1		1	
**Do you think that your experience of having the Flexi-scope test and colonoscopy has made you feel more able to meet your home/work responsibilities?**	**1**,**000 (74.3)**	**184 (18.4)**		0.92		0.92
Not at all	593 (59.3)	111 (18.7)	0.96 (0.59–1.55)		0.97 (0.60–1.57)	
A little bit	121 (12.1)	22 (18.2)	0.92 (0.49–1.75)		0.93 (0.49–1.76)	
Quite a bit	157 (15.7)	26 (16.6)	0.83 (0.45–1.51)		0.83 (0.45–1.52)	
A great deal	129 (12.9)	25 (19.4)	1		1	
**Do you think that your experience of having the Flexi-scope test and colonoscopy has made you sleep better?**	**998 (74.1)**	**187 (18.7)**		0.53		0.54
Not at all	652 (65.3)	119 (18.3)	1.19 (0.67–2.10)		1.20 (0.68–2.12)	
A little bit	130 (13.0)	30 (23.1)	1.59 (0.81–3.12)		1.60 (0.81–3.13)	
Quite a bit	115 (11.5)	22 (19.1)	1.26 (0.62–2.55)		1.26 (0.62–2.55)	
A great deal	101 (10.1)	16 (15.8)	1		1	
**Did you feel anxious when a polyp(s) was found?**	**1**,**064 (79.0)**	**195 (18.3)**		0.81		0.79
Not at all	95 (8.9)	19 (20.0)	1		1	
Somewhat	279 (26.2)	46 (16.5)	0.79 (0.44–1.43)		0.79 (0.44–1.43)	
Moderately	318 (29.9)	59 (18.6)	0.91 (0.51–1.62)		0.92 (0.52–1.64)	
Very	372 (35.0)	71 (19.1)	0.94 (0.54–1.66)		0.95 (0.54–1.68)	
**Did you feel anxious when you were asked to return for a colonoscopy?**	**1**,**062 (78.9)**	**193 (18.2)**		0.50		0.46
Not at all	155 (14.6)	31 (20.0)	1		1	
Somewhat	397 (37.4)	77 (19.4)	0.96 (0.60–1.53)		0.95 (0.60–1.52)	
Moderately	253 (23.8)	46 (18.2)	0.89 (0.54–1.48)		0.89 (0.53–1.48)	
Very	257 (24.2)	39 (15.2)	0.72 (0.43–1.20)		0.69 (0.41–1.18)	
**Did you feel anxious waiting for your colonoscopy appointment?**	**1**,**055 (78.4)**	**192 (18.2)**		0.91		0.89
Not at all	279 (26.4)	49 (17.6)	1		1	
Somewhat	345 (32.7)	65 (18.8)	1.09 (0.72–1.64)		1.10 (0.73–1.65)	
Moderately	269 (25.5)	51 (19.0)	1.10 (0.71–1.69)		1.10 (0.71–1.70)	
Very	162 (15.4)	27 (16.7)	0.94 (0.56–1.57)		0.93 (0.55–1.57)	
**Did you feel anxious whilst waiting in the clinic for your colonoscopy?**	**1**,**058 (78.6)**	**193 (18.2)**		0.52		0.50
Not at all	288 (27.2)	48 (16.7)	1		1	
Somewhat	338 (31.9)	62 (18.3)	1.12 (0.74–1.70)		1.13 (0.75–1.71)	
Moderately	261 (24.7)	55 (21.1)	1.33 (0.87–2.05)		1.35 (0.87–2.08)	
Very	171 (16.2)	28 (16.4)	0.98 (0.59–1.63)		0.98 (0.58–1.66)	
**Did you feel anxious after the colonoscopy whilst waiting for the results?**	**1**,**050 (78.0)**	**192 (18.3)**		0.47		0.46
Not at all	217 (20.7)	43 (19.8)	1		1	
Somewhat	357 (34.0)	58 (16.2)	0.78 (0.51–1.21)		0.79 (0.51–1.22)	
Moderately	250 (23.8)	52 (20.1)	1.06 (0.68–1.67)		1.07 (0.68–1.69)	
Very	226 (21.5)	39 (17.3)	0.84 (0.52–1.36)		0.85 (0.52–1.38)	
**Did you feel anxious after receiving the results of the colonoscopy?** ^**h**^	**1**,**045 (77.6)**	**192 (18.4)**		0.17		0.14
Not at all	745 (71.3)	133 (17.9)	1		1	
Somewhat	162 (15.5)	27 (16.7)	0.92 (0.58–1.45)		0.86 (0.54–1.37)	
Moderately	90 (8.6)	17 (18.9)	1.07 (0.61–1.88)		0.95 (0.54–1.70)	
Very	48 (4.6)	15 (31.3)	2.09 (1.10–3.96)		2.11 (1.10–4.04)	
**Did you have any further bowel examinations after your colonoscopy?** ^**i**^	**1**,**059 (78.9)**	**193 (18.2)**		0.67		0.51
Yes	59 (5.6)	12 (20.3)	1.16 (0.60–2.22)		0.79 (0.39–1.61)	
No	1,000 (94.4)	181 (18.1)	1		1	
**Did you have any problems after the colonoscopy?**	**921 (68.4)**	**172 (18.7)**		0.52		0.55
Yes	156 (16.9)	32 (20.5)	1.15 (0.75–1.77)		1.14 (0.74–1.76)	
No	765 (83.1)	140 (18.3)	1			
**Have you had any other medical problems since the colonoscopy?**	**863 (64.1)**	**161 (18.7)**		0.29		0.29
Yes	71 (8.2)	10 (14.1)	0.70 (0.35–1.39)		0.70 (0.35–1.39)	
No	792 (91.8)	151 (19.1)	1		1	
**Having the tests took up too much time?** ^**j**^	**1**,**053 (78.2)**	**193 (18.3)**		0.27		0.34
Strongly disagree	525 (49.9)	86 (16.4)	1		1	
Disagree	466 (44.3)	93 (20.0)	1.27 (0.92–1.76)		1.26 (0.91–1.75)	
Not sure	21 (2.0)	7 (33.3)	2.55 (1.00-6.51)		2.31 (0.89–6.02)	
Agree	21 (2.0)	3 (14.3)	0.85 (0.25–2.95)		0.75 (0.21–2.64)	
Strongly agree	20 (1.9)	4 (20.0)	1.28 (0.42–3.91)		1.21 (0.39–3.76)	
**The tests made me worry about cancer?**	**1**,**045 (77.6)**	**191 (18.3)**		0.06		0.06
Strongly disagree	173 (16.6)	24 (13.9)	1		1	
Disagree	363 (34.7)	64 (17.6)	1.33 (0.80–2.21)		1.36 (0.81–2.26)	
Not sure	169 (16.2)	42 (24.9)	2.05 (1.18–3.57)		2.10 (1.20–3.66)	
Agree	288 (27.6)	48 (16.7)	1.24 (0.73–2.11)		1.27 (0.75–2.17)	
Strongly agree	52 (5.0)	13 (25.0)	2.07 (0.97–4.43)		2.10 (0.98–4.49)	
**Having the tests was tempting fate?**	**1**,**048 (77.9)**	**189 (18.0)**		0.005		0.005
Strongly disagree	599 (57.2)	89 (14.9)	1		1	
Disagree	349 (33.3)	71 (20.3)	1.46 (1.04–2.07)		1.47 (1.04–2.08)	
Not sure	65 (6.2)	19 (29.2)	2.37 (1.33–4.23)		2.39 (1.34–4.28)	
Strongly agree/agree	35 (3.3)	10 (28.6)	2.29 (1.06–4.94)		2.33 (1.08–5.03)	
**I would rather have let nature take its course?**	**1**,**053 (78.2)**	**192 (18.2)**		0.009		0.009
Strongly disagree	659 (62.6)	107 (16.2)	1		1	
Disagree	329 (31.2)	63 (19.1)	1.22 (0.87–1.72)		1.22 (0.86–1.72)	
Not sure	34 (3.2)	10 (29.4)	2.15 (1.00-4.62)		2.16 (1.00-4.65)	
Strongly agree/agree	31 (2.9)	12 (38.7)	3.26 (1.54–6.91)		3.22 (1.51–6.83)	
**Having the tests made me anxious?** ^**k**^	**1**,**043 (77.5)**	**189 (18.1)**		0.42		0.46
Strongly disagree	161 (15.4)	22 (13.7)	1		1	
Disagree	303 (28.9)	52 (17.2)	1.31 (0.76–2.25)		1.31 (0.76–2.26)	
Not sure	141 (13.5)	29 (20.6)	1.64 (0.89-3.00)		1.58 (0.86–2.93)	
Agree	384 (36.7)	76 (19.9)	1.57 (0.94–2.64)		1.55 (0.92–2.62)	
Strongly agree	58 (5.5)	10 (17.5)	1.34 (0.59–3.04)		1.17 (0.51–2.70)	
**I regret having had tests in that part of the body?**	**1**,**055 (78.4)**	**191 (18.1)**		0.037		0.038
Strongly disagree	620 (58.8)	101 (16.3)	1		1	
Disagree	374 (35.5)	77 (20.6)	1.33 (0.96–1.85)		1.33 (0.96–1.86)	
Not sure	21 (2.0)	8 (38.1)	3.16 (1.28–7.83)		3.14 (1.27–7.78)	
Strongly agree/agree	40 (3.8)	5 (12.5)	0.73 (0.28–1.92)		0.73 (0.28–1.92)	
**I don’t feel I need the tests?**	**1**,**050 (78.0)**	**193 (18.2)**		< 0.001		< 0.001
Strongly disagree	566 (53.9)	80 (14.1)	1		1	
Disagree	391 (37.2)	81 (20.7)	1.59 (1.13–2.23)		1.59 (1.13–2.23)	
Not sure	61 (5.8)	21 (34.4)	3.19 (1.79–5.69)		3.21 (1.80–5.74)	
Strongly agree/agree	32 (3.0)	9 (28.1)	2.38 (1.06–5.32)		2.38 (1.06–5.33)	

Abbreviations: AC = ascending colon. CI = confidence interval. CM = caecum. CRC = colorectal cancer. DC = descending colon. HF = hepatic flexure. OR = Odds ratio. RM = rectum. RS = recto sigmoid. SC = sigmoid colon. SD = sigmoid descending. SF = splenic flexure. TC = transverse colon. TI = terminal ileum

^a^All n and percentage except the entry for age, which is median and interquartile range

^b^ Calculated with the likelihood ratio test

^c^A separate multivariable model was constructed for each specified variable, which included age and sex, the specified variable, and any of the variables family history, bowel preparation quality, or segment of the bowel reached that were classified as having a confounding effect, identified using a one variable in one variable out approach to determine which variables altered the risk estimates by $\:\ge\:$10%. The post-colonoscopy questionnaire comprised of a large amount of questions, where several of the questions were interrelated; due to the risk of collinearity additional questionnaire variables were not assessed for inclusion in multivariable models. For confounding variables, a missing category was created for those with missing values

^d^Satisfaction with way results of colonoscopy explained multivariable model also includes segment of the bowel reached

^e^Tests gave peace of mind multivariable model also includes segment of the bowel reached

^f^Reassured by tests multivariable model also includes segment of the bowel reached

^g^Doing something positive about health multivariable model also includes segment of the bowel reached

^h^Anxious after receiving colonoscopy results multivariable model also includes segment of the bowel reached

^i^Had further bowel exams multivariable model also includes segment of the bowel reached

^j^Tests took up too much time multivariable model also includes segment of the bowel reached

^k^Having tests made anxious multivariable model also includes segment of the bowel reached

**Table 4:** Distal CRC incidence by patient factors, bowel preparation quality, segment of the bowel reached, and questionnaire responses (*n* = 40,141)

	Attended baseline FS *n* (%)^a^	Distal CRC cases *n* (%)^a^	Incidence rate per 100,000 person-years (95%CI)	Univariable HR (95%CI)	*p*-value^b^	Multivariable HR (95%CI)^c^	*p*-value^b^
**Total**	**40**,**141 (100)**	**198 (0.5)**	31.5 (27.4–36.2)				
**Age**,** years**	**60.4 (58.0-62.9)**	**60.7 (58.2–63.0)**		1.04 (0.99–1.09)	0.12	-	-
**Sex**	**40**,**141 (100)**	**198 (0.5)**			< 0.001		-
Male	20,223 (50.4)	122 (0.6)	39.4 (33.0-47.1)	1		-	
Female	19,918 (49.6)	76 (0.4)	23.8 (19.0-29.9)	0.59 (0.45–0.79)		-	
**Family history of CRC**	**37**,**604 (93.7)**	**185 (0.5)**			0.007		0.006
No	33,229 (88.4)	151 (0.5)	29.2 (24.9–34.2)	1		1	
Yes	4,375 (11.6)	34 (0.8)	50.1 (35.8–70.1)	1.73 (1.91–2.51)		1.75 (1.20–2.54)	
**Bowel preparation quality at first FS** ^**d**^	**39**,**390 (98.1)**	**191 (0.5)**			0.038		0.25
Excellent	15,965 (40.5)	65 (0.4)	25.7 (20.1–32.7)	1		1	
Good	13,093 (33.2)	62 (0.5)	30.3 (23.6–38.9)	1.19 (0.84–1.68)		1.17 (0.82–1.65)	
Adequate	7,809 (19.8)	44 (0.6)	36.6 (27.2–49.2)	1.44 (0.99–2.12)		1.38 (0.94–2.04)	
Poor	2,523 (6.4)	20 (0.8)	51.6 (33.3–79.9)	2.03 (1.23–3.36)		1.63 (0.88–2.99)	
**Segment reached at first FS** ^**e**^	**39**,**718 (98.9)**	**194 (0.5)**			0.25		0.31
RM/RS/SC	5,735 (14.4)	35 (0.6)	39.6 (28.4–55.2)	1		1	
SD	7,549 (19.0)	31 (0.4)	26.2 (18.4–37.2)	0.66 (0.40–1.06)		0.71 (0.41–1.23)	
DC	21,507 (54.1)	109 (0.5)	32.2 (26.7–38.9)	0.81 (0.55–1.18)		0.79 (0.50–1.25)	
SF/ TC/ HF/ AC/CM/TI	4,927 (12.4)	19 (0.4)	24.7 (15.8–38.8)	0.62 (0.36–1.09)		0.57 (0.30–1.06)	
**Post first FS questionnaire responses**							
**Test pain**	**38**,**990 (97.1)**	**191 (0.5)**			0.026		0.09
None	10,612 (27.2)	66 (0.6)	39.8 (31.3–50.6)	1		1	
Mild	20,542 (52.7)	98 (0.5)	30.5 (25.0-37.2)	0.77 (0.56–1.05)		0.80 (0.59–1.10)	
Quite a lot/severe	7,836 (20.1)	27 (0.3)	22.0 (15.0–32.0)	0.55 (0.35–0.87)		0.61 (0.39–0.97)	
**Expected pain**	**38**,**673 (96.3)**	**189 (0.5)**			0.047		0.012
Less painful	16,484 (42.6)	101 **(**0.6**)**	39.3 (32.4–47.8)	1		1	
About the same	14,564 (37.7)	63 (0.4)	27.5 (21.5–35.2)	0.70 (0.51–0.95)		0.71 (0.52–0.97)	
More painful	7,625 (19.7)	25 (0.3)	20.9 (14.1–30.9)	0.53 (0.34–0.82)		0.57 (0.37–0.88)	
**Abdominal pain or cramps**	**35**,**988 (89.7)**	**175 (0.5)**			0.022		0.05
No symptoms	24,129 (67.0)	131 (0.5)	34.7 (29.2–41.2)	1		1	
Any symptoms	11,859 (33.0)	44 (0.4)	23.6 (17.6–31.7)	0.68 (0.48–0.95)		0.71 (0.51–1.01)	
**Nausea or vomiting** ^**f**^	**33**,**488 (83.4)**	**163 (0.5)**			0.93		0.52
No symptoms	32,011 (95.6)	156 (0.5)	31.1 (26.6–36.4)	1		1	
Any symptoms	1,477 (4.4)	7 (0.5)	30.1 (14.4–63.2)	0.97 (0.45–2.06)		1.35 (0.56–3.27)	
**Faintness or dizziness** ^**g**^	**33**,**716 (84.0)**	**165 (0.5)**			0.53		0.98
No symptoms	30,763 (91.2)	153 (0.5)	31.7 (27.0-37.1.)	1		1	
Any symptoms	2,953 (8.8)	12 (0.4)	26.2 (14.9–46.2)	0.83 (0.46–1.50)		0.99 (0.53–1.87)	
**Wind** ^**h**^	**37**,**286 (92.9)**	**183 (0.5)**			0.51		0.41
None	14,124 (37.9)	66 (0.5)	29.8 (23.4–38.0)	1		1	
Mild	15,611 (41.9)	84 (0.5)	34.4 (27.8–42.6)	1.15 (0.83–1.59)		1.26 (0.90–1.75)	
Moderate/severe	7,551 (20.3)	33 (0.4)	27.9 (19.8–39.2)	0.93 (0.61–1.41)		1.16 (0.74–1.82)	
**Bottom soreness**	**34**,**864 (86.9)**	**166 (0.5)**			0.30		0.31
No symptoms	24,324 (69.8)	122 (0.5)	32.0 (26.0-26.8)	1		1	
Any symptoms	10,540 (30.2)	44 (0.4)	26.7 (19.8–35.8)	0.83 (0.59–1.18)		0.84 (0.59–1.19)	
**Soiling**	**33**,**713 (84.0)**	**168 (0.5)**			0.31		0.31
No symptoms	29,927 (88.8)	145 (0.5)	30.9 (26.2–36.3)	1		1	
Any symptoms	3,786 (11.2)	23 (0.6)	39.1 (26.0-58.8)	1.27 (0.82–1.97)		1.26 (0.81–1.96)	
**Sleep disturbance** ^**i**^	**33**,**748 (84.1)**	**168 (0.5)**			0.37		0.55
No symptoms	30,467 (90.3)	155 (0.5)	32.4 (27.7–38.0)	1		1	
Any symptoms	3,281 (9.7)	13 (0.4)	25.3 (14.7–43.5)	0.78 (0.44–1.37)		0.84 (0.46–1.52)	

Abbreviations: AC = ascending colon. CI = confidence interval. CM = caecum. CRC = colorectal cancer. DC = descending colon. FS = flexible sigmoidoscopy. HF = hepatic flexure. HR = hazard ratio. RM = rectum. RS = recto sigmoid. SC = sigmoid colon. SD = sigmoid descending. SF = splenic flexure. TC = transverse colon. TI = terminal ileum

^a^All n and percentage except the entry for age, which is median and interquartile range

^b^ Calculated with the likelihood ratio test

^c^A separate multivariable model was constructed for each specified variable, which included age, sex, the specified variable, and any additional variables classified as having a confounding effect, identified using a one variable in, one variable out approach to determine which variables altered the risk estimates by $\:\ge\:$10%. For confounding variables, a missing category was created for those with missing values, although it was necessary to exclude further participants from some multivariable models due to a lack of events in the missing category

^d^Bowel preparation quality multivariable model also includes segment of the bowel reached

^e^Segment of the bowel reached multivariable model also includes expected pain and bowel preparation quality

^f^Nausea or vomiting multivariable model also includes expected pain, abdominal pain or cramps, and faintness or dizziness (participants missing data on expected pain excluded, *n* = 32,929 included)

^g^Faintness or dizziness multivariable model also includes expected pain and abdominal pain or cramps (participants missing data on expected pain excluded, *n* = 33,149 included)

^h^Wind multivariable model also includes test pain and abdominal pain or cramps (participants missing data on test pain excluded, *n* = 36,942 included)

^i^Sleep disturbance multivariable model also includes abdominal pain or cramps

In comparison to reaching the caecum/terminal ileum at colonoscopy, only reaching more distally was associated with increased odds of non-attendance at surveillance (multivariable: OR 2.06, 95%CI 1.33–3.20). In addition, participants who did not strongly agree that they had made the right decision to take the tests had higher odds of non-attendance for surveillance (multivariable: strongly disagree/disagree OR 5.28, 95%CI 1.50-18.56; not sure OR 3.74, 95%CI 1.39–10.04; agree OR 1.44, 95%CI 1.04–2.01, all vs. strongly agree). Compared to those who strongly disagreed, those who agreed or strongly agreed that having the tests was tempting fate had higher odds of non-attendance at surveillance (multivariable: OR 2.33, 95%CI 1.08–5.03).

Participants who strongly agreed/agreed that they would rather have let nature take its course had higher odds of non-attendance for surveillance (multivariable: OR 3.22, 95%CI 1.51–6.83, vs. strongly disagree). Those who agreed or strongly agreed with the statement ‘I don’t feel I need the tests’ had increased odds of non-attendance compared to those who strongly disagreed with this statement (multivariable: OR 2.38, 95%CI 1.06–5.33; Table 3).

### CRC incidence

Of the 40,141 eligible participants who had a baseline FS, the median age at FS was 60.4 years, 50.4% were males, and 11.6% had at least one first degree relative with CRC (Table 4). During a median of 16.8 years follow-up, 198 (0.5%) participants were diagnosed with distal CRC, giving an incidence rate of 31.5 per 100,000 person-years (95%CI 27.4–36.2).

Females had a decreased risk of distal CRC compared to males (HR 0.59, 95% CI 0.45–0.79) (Table 4 and Supplementary Fig. 1A). A family history of CRC was positively associated with distal CRC (yes vs. no: multivariable HR 1.75, 95%CI 1.20–2.54) (Table 4 and Supplementary Fig. 1B). Bowel preparation quality, segment reached, and test pain were not associated with distal CRC in multivariable models (Table 4 and Supplementary Fig. 1 C, D, E).

Individuals who reported the baseline FS to be about as painful or more painful than expected had a decreased risk of distal CRC compared to those who considered it less painful (multivariable: HR 0.71, 95%CI 0.52–0.97; HR 0.57, 95%CI 0.37–0.88, respectively) (Table 4 and Supplementary Fig. 1F). In addition, those who reported abdominal pain/cramps had a lower risk of distal CRC compared to those without these symptoms (multivariable: HR 0.71, 95%CI 0.51–1.01) (Table 4 and Supplementary Fig. 1G), although this was borderline significant. Nausea/vomiting, faintness/dizziness, wind, bottom soreness, soiling, and sleep disturbance were not associated with distal CRC (Table 4 and Supplementary Fig. 1 H-M). We investigated if experiencing pain and symptoms at/after FS was associated with total procedure time and found that all post first FS questionnaire responses, except for nausea/vomiting, were associated with a longer baseline FS procedure time (Supplementary Table 10).

## Discussion

We investigated the impact of patient experience of endoscopic screening on attendance at future examinations and on distal CRC incidence. Patient experience during baseline endoscopic examinations may influence attendance at subsequent examinations; therefore, it is essential that a patient’s experience be optimised to increase the likelihood of future attendance. An individual’s experience of an endoscopic examination could affect CRC incidence by impacting on their willingness to attend future examinations, including those needed due to experiencing symptoms or participating in a screening programme, over the long term. We found that reaching only the rectosigmoid section of the bowel and symptoms of faintness/dizziness were associated with non-attendance at repeat FS. Non-attendance at surveillance was increased when baseline colonoscopy had not reached the caecum/terminal ileum and was associated with whether participants felt that they had made the right decision to take the tests, that they needed the tests, that they would rather have let nature take its course, or that taking the tests was tempting fate. Family history of CRC was positively associated with distal CRC, whereas having a FS that was more painful than expected was inversely associated.

A FS with a 60 cm maximum scope insertion distance can potentially reach the splenic flexure or further [28]. It is important for the sigmoidoscope to reach as high as possible as examining a greater surface area of the colonic mucosa increases the efficacy of the examination [27, 29]. In our FS analyses, the majority of examinations reached at least the sigmoid colon or more proximally (72.5%). Participants whose FS reached only the rectosigmoid junction were at increased risk of non-attendance at repeat FS. We also observed an increase in non-attendance at surveillance colonoscopy when baseline colonoscopy examinations failed to reach the caecum/terminal ileum. A colonoscopy can reach as far as the caecum [30].

Technically inadequate FS examinations (insertion of the scope < 50 cm or < 90% of the mucosal surface is viewed) have been associated with female sex and increasing age, with most being due to patient discomfort [27]; all of these were corroborated in our data. Previous research reported that failing to reach the optimum section of the bowel was associated with increased pain [18] and pain has been shown to be a key factor in non-attendance [8, 10, 14]. In the UKFSST, the model of endoscope used during FS procedures was likely less flexible with a wider diameter compared to those currently used, which may have contributed to increased feelings of pain. We found no association between pain experienced at FS and non-attendance at repeat FS or baseline colonoscopy; however, we did not have data on pain experienced at colonoscopy. Odds ratios for non-attendance at repeat FS generally tended to show an increase in risk with increasing pain but there was a limited number of cases $\:(<$10) for the highest category of each of the pain variables, contributing to a lack of statistical significance. Poor bowel cleansing can result in incomplete examinations [31, 32] and longer and more difficult procedures [33]. In our FS analyses, the majority (79.9%) of repeat FS examinations were due to poor bowel preparation. Bowel preparation quality is modifiable and improving the quality at the first examination could reduce the chance of an incomplete exam and the need for repeat examination, thus ameliorating the risk of non-attendance. In our study, participants used a single phosphate enema (Fletchers’ phosphate enema, long tube version for self-administration, Pharmax Ltd, Bexley, Kent), provided along with instructions for use [21]. Improving a patient’s knowledge on how to adequately cleanse the bowel would not only benefit the patient but could reduce the level of difficulty for the endoscopist. However, in our study, bowel preparation quality was not associated with non-attendance in multivariable models.

We showed that individuals who experienced post-exam faintness/dizziness had increased odds of non-attendance at repeat FS but not at baseline colonoscopy. Experiencing these symptoms likely negatively impacts on an individual’s overall satisfaction with the examination. Ensuring individuals are aware of potential side effects and the short-term cost of these against the long-term benefits of an examination could make side effects more tolerable, aiding future attendance.

An individual not agreeing strongly they had made the right decision to take the test was associated with non-attendance at surveillance. Previous research has reported that individuals with or without prior experience of endoscopy are willing to overcome unpleasantness and embarrassment associated with the invasive nature of the exam to gain reassurance from the test [34]. But experiencing negative feelings associated with examination could prompt individuals to reflect and reconsider whether they had made the right decision to undertake the tests, thus affecting their decision to repeat the examination in the future.

Fatalism has previously been reported as a barrier to screening [20, 35] and is a belief held by individuals about the presence of cancer likely resulting in death [36]. Our results support this, as we found that participants who felt that having the tests was tempting fate or who agreed they would rather have let nature take its course had an increased risk of non-attendance at surveillance. Identifying if fatalism is present would offer the opportunity to implement strategies to modify this belief and to educate patients that screening and surveillance have the capacity to change a person’s future risk.

A person’s perceived risk of CRC could influence endoscopy attendance. Even when individuals agreed with the benefits of endoscopic screening for the early detection of CRC, this knowledge did not alter their own perceived risk [34]. Even though a family history of CRC is known to increase the risk of this disease [37], it has been reported that those with such family history tended to underestimate their own risk, even though they recognised this as a risk factor [38]. We found that individuals who felt that they did not need the test had higher odds of non-attendance at surveillance. This feeling of a lack of need to attend an examination could be associated with a person’s beliefs about their perceived risk, which could be driven by an absence of concerning symptoms or because they consider their lifestyle to be healthy [34]. Previous research involving individuals who were classed as high-risk after resection of a large ($\:\ge\:$1 cm) adenoma reported that an absence of symptoms was a reason for non-compliance [39].

We found that FS examinations that were as painful as expected, more painful than expected or resulting in symptoms of abdominal pain/cramps were associated with a decreased risk of distal CRC; further investigations showed that they were also associated with longer procedure times. These symptoms could be the result of endoscope looping and/or manual pressure during the procedure [40] or longer procedure times due to the removal of abnormalities [41]. Specifically, the removal of polyps likely explains the lower CRC risk associated with symptoms of increased pain. Compared with current endoscopic screening methods, our study participants were not offered sedation and a different model of endoscope was used during the examination, which may have increased the likelihood of experiencing pain. Offering individuals adequate pain relief may ease discomfort, or sedation could improve the patient experience.

A strength of our study is that it uses a large high-quality dataset. Our study differs from previous research as it includes asymptomatic patients who informed on their experience at an endoscopic examination along with endoscopist reported examination variables; this allowed for an understanding of the factors affecting non-attendance at future examinations in an average risk population, with 17 years of follow-up. Limitations include the lack of data on patient experience of bowel preparation, which is an important aspect of the examination and may influence other factors, and having a six-month gap to completion of post-colonoscopy questionnaires, which could result in recall bias. A further limitation is the low non-attendance rate at referred colonoscopy (3.4%), which resulted in a lack of statistical power; this low rate is likely due to participants being informed they were higher risk, increasing their motivation to attend colonoscopy. There was also a lack of statistical power to investigate the effects of non-attendance at repeat FS, referred colonoscopy, and surveillance on distal CRC incidence. Additionally, the timespan between first FS and repeat FS or referred colonoscopy is far shorter than previous studies reporting on non-adherence at endoscopic examinations [9, 10]; the UKFSST protocol aimed to offer participants a repeat FS examination or referred colonoscopy as soon as possible. Finally, this study population was a selective group, which could limit generalisability to other situations.

In conclusion, high patient adherence with screening and surveillance is essential to realise the full benefits on CRC prevention and early detection [7]. We identified several factors associated with non-attendance at future endoscopic examinations and distal CRC incidence. Experiencing an exam that did not reach the ideal depth of insertion and existing thoughts and beliefs were important contributing factors to non-attendance at repeat examinations. The association between family history of CRC and incidence of distal CRC highlights the need for individuals to be fully aware of this risk factor. The experience of pain both during and after an examination was associated with lower distal CRC risk. These findings highlight the importance of patients being educated about how to correctly administer bowel preparation, being fully informed about potential post-examination symptoms, and being given the opportunity to discuss their beliefs and thoughts about an endoscopic examination. Using long-term follow-up data has allowed for information to be gained that will be beneficial in optimising current endoscopy-based screening and surveillance programmes. Considering the importance of endoscopic examination, it is vital that patient experience is optimised to increase the likelihood of future attendance.

## Electronic Supplementary Material

Below is the link to the electronic supplementary material.

Supplementary Material 1

## Data Availability

The individual-level data used in this current study is not available as it consists of confidential patient-identifiable data. Requests regarding data should be directed to the corresponding author.
