# Supplementary material for: The impact of patient-reported factors of endoscopic screening experience on attendance at future examinations and distal colorectal cancer incidence
Source: BMC Cancer. 2025 Mar 6;25:409. doi: 10.1186/s12885-025-13771-3 (PMC11887164; doi:10.1186/s12885-025-13771-3)
Supplement: Supplementary file 1 — Supplementary Material 1 [file 12885_2025_13771_MOESM1_ESM.docx]

**Supplementary appendix**

**Supplementary Table 1.** Reasons for referral to repeat flexible sigmoidoscopy.

| **Reason for referral to repeat flexible sigmoidoscopy** | **Referred for same day repeat flexible sigmoidoscopy examination**  **n (%)^a^** | **Referred for later day repeat flexible sigmoidoscopy examination**  **n (%)^a^** |
| --- | --- | --- |
| Poor bowel preparation | 1,312(98.9) | 679 (79.9) |
| Warfarin/aspirin | 1(0.1) | 70 (8.2) |
| Pain | 4(0.3) | 42 (4.9) |
| Check excision site | 0 (0) | 13 (1.5) |
| Find polyps seen previously | 1(0.1) | 7 (0.8) |
| Difficult polypectomy | 0 (0) | 6 (0.7) |
| Difficult flexible sigmoidoscopy | 1(0.1) | 4 (0.5) |
| Technical problem with equipment | 0 (0) | 4 (0.5) |
| Bleeding | 0 (0) | 3 (0.4) |
| Fainting/vasovagal | 1(0.1) | 2 (0.2) |
| Incomplete flexible sigmoidoscopy | 0 (0) | 1 (0.1) |
| Safety of polypectomy | 1(0.1) | 0 (0) |
| Difficult to retain air | 1(0.1) | 0 (0) |
| Opened bowels | 1(0.1) | 0 (0) |
| Unknown | 3 (0.2) | 19 (2.2) |
| **Total** | 1,326 (100) | 850 (100) |

^a^Because of rounding, percentages do not total to 100.0.

**Supplementary Table 2.** Post-flexible sigmoidoscopy examination questionnaire^a^.

|  | **Question** |
| --- | --- |
| 1. | How much pain did you feel during the test?  (No pain, mild pain, quite a lot of pain, severe pain) |
| 2. | Was the test more painful or less painful than expected?  (Less painful than I expected, about the same as I expected, more painful than I expected) |
|  | Did you have any of the following after the test? (None, mild, moderate, severe) |
| 3. | Abdominal pain or cramps |
| 4. | Nausea or vomiting |
| 5. | Faint feeling or dizziness |
| 6. | Wind |
| 7. | Bottom soreness |
| 8. | Soiling |
| 9. | Sleep disturbance |

^a^We excluded an open-ended question which asked the participant to provide other comments about any aspect of the test; a question which asked how they travelled to the examination centre; and a question which asked what day and time of day would have been the most convenient for them to have attended the examination.

**Supplementary Table 3.** Post-colonoscopy examination questionnaire^a^.

|  | **Question** |
| --- | --- |
| 1. | How satisfied were you with the information you were given before your colonoscopy?  (Very satisfied, satisfied, dissatisfied, very dissatisfied) |
| 2. | How satisfied were you with the way the results of the colonoscopy were explained to you?  (Very satisfied, satisfied, dissatisfied, very dissatisfied) |
| 3. | The tests gave me piece of mind?  (Strongly disagree, disagree, not sure, agree, strongly agree) |
| 4. | Having the tests reduced my chance of getting bowel cancer?  (Strongly disagree, disagree, not sure, agree, strongly agree) |
| 5. | I made the right decisions to take the tests?  (Strongly disagree, disagree, not sure, agree, strongly agree) |
| 6. | The tests reassured me?  (Strongly disagree, disagree, not sure, agree, strongly agree) |
| 7. | Having the tests made me feel that I was doing something positive about my health?  (Strongly disagree, disagree, not sure, agree, strongly agree) |
| 8. | A screening test for bowel cancer is important?  (Strongly disagree, disagree, not sure, agree, strongly agree) |
| 9. | Do you think that your experience of having the Flexi-scope test and colonoscopy has made you feel more relaxed?  (Not at all, a little bit, quite a bit, a great deal) |
| 10. | Do you think that your experience of having the Flexi-scope test and colonoscopy has led to improved relationships with friends or relations?  (Not at all, a little bit, quite a bit, a great deal) |
| 11. | Do you think that your experience of having the Flexi-scope test and colonoscopy has made you feel more able to meet your home and/or work responsibilities?  (Not at all, a little bit, quite a bit, a great deal) |
| 12. | Do you think that your experience of having the Flexi-scope test and colonoscopy has made you sleep better?  (Not at all, a little bit, quite a bit, a great deal) |
| 13. | Did you feel anxious when a polyp(s) was found?  (Not at all, somewhat, moderately, very) |
| 14. | Did you feel anxious when you were asked to return for a colonoscopy?  (Not at all, somewhat, moderately, very) |
| 15. | Did you feel anxious waiting for your colonoscopy appointment?  (Not at all, somewhat, moderately, very) |
| 16. | Did you feel anxious whilst waiting in the clinic for your colonoscopy?  (Not at all, somewhat, moderately, very) |
| 17. | Did you feel anxious after the colonoscopy whilst waiting for the results?  (Not at all, somewhat, moderately, very) |
| 18. | Did you feel anxious after receiving the results of the colonoscopy?  (Not at all, somewhat, moderately, very) |
| 19. | Did you have any further examinations after your colonoscopy? (Yes, no) |
| 20. | Did you have any problems after the colonoscopy? (Yes, no) |
| 21. | Have you had any other medical problems since the colonoscopy? (Yes, no) |
| 22. | Having the tests took up too much time?  (Strongly disagree, disagree, not sure, agree, strongly agree) |
| 23. | The tests made me worry about cancer?  (Strongly disagree, disagree, not sure, agree, strongly agree) |
| 24. | Having the tests was tempting fate?  (Strongly disagree, disagree, not sure, agree, strongly agree) |
| 25. | I would rather have let nature take its course?  (Strongly disagree, disagree, not sure, agree, strongly agree) |
| 26. | Having the tests made me anxious?  (Strongly disagree, disagree, not sure, agree, strongly agree) |
| 27. | I regret having had tests in that part of the body?  (Strongly disagree, disagree, not sure, agree, strongly agree) |
| 28. | I don't feel I needed the tests?  (Strongly disagree, disagree, not sure, agree, strongly agree) |

^a^We excluded open-ended questions and questions which asked the participant to provide further information if they responded with ‘yes’ to a question.

**Supplementary Table 4.** Protocol guidelines for the categorisation of bowel preparation quality.

| **Bowel preparation category** | **Definition** |
| --- | --- |
| Excellent | No stool or fluid present. |
| Good | Some residual liquid or stool present but removable by suction, allowing for a completely reliable examination. |
| Adequate | Examination accomplished, but with difficulty due to residual stool. The reliability of the examination is suboptimal. |
| Poor | The examination could not be performed satisfactorily due to the presence of excess faeces. |

**Supplementary Table 5.** Pairwise Pearson’s correlation matrix for the variables included in the analysis of non-attendance at later day repeat FS (n=850)**.**

|  | **Age** | **Sex** | **Family history of colorectal cancer** | **Bowel preparation quality** | **Segment reached** | **Test pain** | **Expected pain** | **Abdominal pain or cramps** | **Nausea or vomiting** | **Faintness or dizziness** | **Wind** | **Bottom soreness** | **Soiling** | **Sleep disturbance** |
| --- | --- | --- | --- | --- | --- | --- | --- | --- | --- | --- | --- | --- | --- | --- |
| **Age** | 1 |  |  |  |  |  |  |  |  |  |  |  |  |  |
| **Sex** | -0.01 | 1 |  |  |  |  |  |  |  |  |  |  |  |  |
| **Family history of colorectal cancer** | -0.05 | -0.02 | 1 |  |  |  |  |  |  |  |  |  |  |  |
| **Bowel preparation quality** | 0.02 | 0.11 | -0.05 | 1 |  |  |  |  |  |  |  |  |  |  |
| **Segment reached** | -0.04 | -0.13 | 0.07 | -0.57 | 1 |  |  |  |  |  |  |  |  |  |
| **Test pain** | -0.01 | 0.11 | 0.02 | -0.23 | 0.12 | 1 |  |  |  |  |  |  |  |  |
| **Expected pain** | -0.01 | 0.07 | 0.04 | -0.14 | 0.06 | 0.67 | 1 |  |  |  |  |  |  |  |
| **Abdominal pain or cramps** | 0.04 | 0.16 | 0.07 | -0.10 | -0.01 | 0.35 | 0.29 | 1 |  |  |  |  |  |  |
| **Nausea or vomiting** | -0.03 | 0.13 | 0.001 | -0.005 | -0.07 | 0.17 | 0.12 | 0.30 | 1 |  |  |  |  |  |
| **Faintness or dizziness** | -0.001 | 0.12 | 0.05 | -0.07 | -0.01 | 0.23 | 0.23 | 0.28 | 0.29 | 1 |  |  |  |  |
| **Wind** | 0.03 | 0.01 | 0.05 | -0.08 | 0.06 | 0.27 | 0.21 | 0.43 | 0.21 | 0.21 | 1 |  |  |  |
| **Bottom soreness** | 0.02 | 0.05 | -0.04 | -0.03 | -0.07 | 0.22 | 0.22 | 0.32 | 0.21 | 0.22 | 0.28 | 1 |  |  |
| **Soiling** | 0.06 | 0.07 | 0.03 | 0.03 | -0.08 | 0.11 | 0.14 | 0.21 | 0.17 | 0.22 | 0.26 | 0.26 | 1 |  |
| **Sleep disturbance** | -0.09 | 0.06 | -0.01 | -0.06 | 0.01 | 0.23 | 0.22 | 0.33 | 0.30 | 0.22 | 0.27 | 0.23 | 0.24 | 1 |

**Abbreviations:** FS=flexible sigmoidoscopy.

**Supplementary Table 6.** Pairwise Pearson’s correlation matrix for variables included in the analysis of non- attendance at referred colonoscopy (n=1,788).

|  | **Age** | **Sex** | **Family history of colorectal cancer** | **Bowel preparation quality** | **Segment reached** | **Test pain** | **Expected pain** | **Abdominal pain or cramps** | **Nausea or vomiting** | **Faintness or dizziness** | **Wind** | **Bottom soreness** | **Soiling** | **Sleep disturbance** |
| --- | --- | --- | --- | --- | --- | --- | --- | --- | --- | --- | --- | --- | --- | --- |
| **Age** | 1 |  |  |  |  |  |  |  |  |  |  |  |  |  |
| **Sex** | 0.05 | 1 |  |  |  |  |  |  |  |  |  |  |  |  |
| **Family history of colorectal cancer** | 0.02 | 0.01 | 1 |  |  |  |  |  |  |  |  |  |  |  |
| **Bowel preparation quality** | 0.04 | -0.03 | 0.01 | 1 |  |  |  |  |  |  |  |  |  |  |
| **Segment reached** | -0.03 | -0.18 | -0.01 | -0.15 | 1 |  |  |  |  |  |  |  |  |  |
| **Test pain** | 0.02 | 0.13 | -0.02 | -0.03 | -0.14 | 1 |  |  |  |  |  |  |  |  |
| **Expected pain** | 0.02 | 0.12 | -0.04 | -0.03 | -0.13 | 0.70 | 1 |  |  |  |  |  |  |  |
| **Abdominal pain or cramps** | -0.03 | 0.14 | 0.03 | -0.05 | -0.01 | 0.33 | 0.28 | 1 |  |  |  |  |  |  |
| **Nausea or vomiting** | -0.03 | 0.14 | 0.02 | 0.03 | -0.06 | 0.14 | 0.12 | 0.23 | 1 |  |  |  |  |  |
| **Faintness or dizziness** | -0.05 | 0.09 | 0.01 | 0.04 | -0.10 | 0.14 | 0.13 | 0.20 | 0.29 | 1 |  |  |  |  |
| **Wind** | -0.04 | 0.04 | -0.02 | -0.01 | -0.01 | 0.21 | 0.16 | 0.43 | 0.12 | 0.14 | 1 |  |  |  |
| **Bottom soreness** | -0.02 | 0.05 | 0.01 | -0.03 | -0.05 | 0.23 | 0.22 | 0.28 | 0.09 | 0.17 | 0.26 | 1 |  |  |
| **Soiling** | -0.04 | 0.03 | 0.01 | 0.02 | -0.02 | 0.11 | 0.10 | 0.15 | 0.08 | 0.09 | 0.17 | 0.20 | 1 |  |
| **Sleep disturbance** | 0.04 | 0.11 | 0.03 | -0.01 | -0.06 | 0.17 | 0.17 | 0.31 | 0.21 | 0.22 | 0.25 | 0.20 | 0.17 | 1 |

**Supplementary Table 7.** Pairwise Pearson’s correlation matrix for variables included in the analysis of distal colorectal cancer incidence (n=40,141).

|  | **Age** | **Sex** | **Family history of colorectal cancer** | **Bowel preparation quality** | **Segment reached** | **Test pain** | **Expected pain** | **Abdominal pain or cramps** | **Nausea or vomiting** | **Faintness or dizziness** | **Wind** | **Bottom soreness** | **Soiling** | **Sleep disturbance** |
| --- | --- | --- | --- | --- | --- | --- | --- | --- | --- | --- | --- | --- | --- | --- |
| **Age** | 1 |  |  |  |  |  |  |  |  |  |  |  |  |  |
| **Sex** | 0.001 | 1 |  |  |  |  |  |  |  |  |  |  |  |  |
| **Family history of colorectal cancer** | 0.01 | 0.02 | 1 |  |  |  |  |  |  |  |  |  |  |  |
| **Bowel preparation quality** | 0.04 | -0.04 | 0.002 | 1 |  |  |  |  |  |  |  |  |  |  |
| **Segment reached** | -0.04 | -0.20 | -0.01 | -0.27 | 1 |  |  |  |  |  |  |  |  |  |
| **Test pain** | 0.01 | 0.17 | 0.01 | 0.001 | -0.18 | 1 |  |  |  |  |  |  |  |  |
| **Expected pain** | 0.002 | 0.11 | 0.0004 | 0.01 | -0.16 | 0.68 | 1 |  |  |  |  |  |  |  |
| **Abdominal pain or cramps** | -0.02 | 0.14 | 0.02 | 0.01 | -0.06 | 0.26 | 0.21 | 1 |  |  |  |  |  |  |
| **Nausea or vomiting** | 0.0003 | 0.12 | -0.000 | 0.02 | -0.05 | 0.12 | 0.11 | 0.20 | 1 |  |  |  |  |  |
| **Faintness or dizziness** | 0.006 | 0.08 | 0.01 | 0.03 | -0.07 | 0.14 | 0.13 | 0.19 | 0.30 | 1 |  |  |  |  |
| **Wind** | 0.01 | 0.04 | 0.02 | 0.02 | -0.04 | 0.20 | 0.16 | 0.38 | 0.14 | 0.13 | 1 |  |  |  |
| **Bottom soreness** | 0.01 | 0.02 | 0.01 | 0.05 | -0.07 | 0.17 | 0.14 | 0.20 | 0.10 | 0.12 | 0.20 | 1 |  |  |
| **Soiling** | 0.03 | 0.01 | 0.01 | 0.05 | -0.05 | 0.09 | 0.10 | 0.14 | 0.10 | 0.09 | 0.24 | 0.17 | 1.00 |  |
| **Sleep disturbance** | 0.01 | 0.06 | 0.01 | 0.003 | -0.03 | 0.13 | 0.12 | 0.22 | 0.17 | 0.18 | 0.22 | 0.16 | 0.14 | 1 |

**Supplementary Table 8.** First referred procedure by patient factors, bowel preparation quality, segment of the bowel reached and questionnaire responses^a^.

|  | **Not referred for repeat FS, colonoscopy, barium enema or surgery**  **n (%)^b^** | **Referred for later day repeat FS**  **n (%)^b^** | **p-value^c^** | **Referred for colonoscopy**  **n (%)^b,d^** | **p-value^e^** |
| --- | --- | --- | --- | --- | --- |
| **Total** | **36,123 (100)** | **850 (100)** | **-** | **1,788 (100)** | **-** |
| **Age, years** | **60.4 (58.0-62.8)** | **60.7 (58.1-63.0)** | **-** | **60.8 (58.2-63.3)** | **-** |
| **Sex** | **36,123 (100)** | **850 (100)** | **<0.001** | **1,660 (92.8)** | **<0.001** |
| Male | 17,787 (49.2) | 478 (56.2) |  | 1,219 (68.2) |  |
| Female | 18,336 (50.8) | 372 (43.8) |  | 569 (31.8) |  |
| **Family history of CRC** | **33,847 (93.7)** | **812 (95.5)** | **0.052** | **1,660 (92.8)** | **<0.001** |
| No | 30,005 (88.6) | 702 (86.5) |  | 1,419 (85.5) |  |
| Yes | 3,842 (11.4) | 110 (13.5) |  | 241 (14.5) |  |
| **Bowel preparation quality at FS** | **35,501 (98.3)** | **839 (98.7)** | **<0.001** | **1,687 (94.4)** | **<0.001** |
| Excellent | 15,186 (42.8) | 61 (7.3) |  | 694 (41.1) |  |
| Good | 12,440 (35.0) | 52 (6.2) |  | 571 (33.8) |  |
| Adequate | 7,262 (20.5) | 151 (18.0) |  | 340 (20.2) |  |
| Poor | 613 (1.7) | 575 (68.5) |  | 82 (4.9) |  |
| **Segment reached at FS** | **35,824 (99.2)** | **812 (95.5)** | **<0.001** | **1,752 (98.0)** | **<0.001** |
| RM | 33 (0.1) | 91 (11.2) |  | 5 (0.3) |  |
| RS | 186 (0.5) | 132 (16.3) |  | 19 (1.1) |  |
| SC | 3,501 (9.8) | 341 (42.0) |  | 227 (13.0) |  |
| SD | 7,179 (20.0) | 41 (5.0) |  | 292 (16.7) |  |
| DC | 20,289 (56.6) | 184 (22.7) |  | 947 (54.1) |  |
| SF/TC/HF/AC/CM/TI | 4,636 (12.9) | 23 (2.8) |  | 262 (15.0) |  |
| **Post first FS questionnaire responses** |  |  |  |  |  |
| **Test pain** | **35,217 (97.5)** | **715 (84.1)** | **<0.001** | **1,731 (96.8)** | **0.12** |
| None | 9,492 (27.0) | 288 (40.3) |  | 502 (29.0) |  |
| Mild | 18,599 (52.8) | 331 (46.3) |  | 902 (52.1) |  |
| Quite a lot/severe | 7,126 (20.2) | 96 (13.4) |  | 327 (18.9) |  |
| **Expected pain** | **34,926 (96.7)** | **704 (82.8)** | **<0.001** | **1,725 (96.5)** | **0.74** |
| Less painful | 14,892 (42.6) | 351 (50.0) |  | 741 (43.0) |  |
| About the same | 13,178 (37.7) | 251 (35.7) |  | 636 (36.9) |  |
| More painful | 6,856 (19.6) | 102 (14.5) |  | 348 (20.2) |  |
| **Abdominal pain or cramps** | **32,582 (90.2)** | **621 (73.1)** | **0.34** | **1,582 (88.5)** | **0.85** |
| None | 21,913 (67.3) | 431 (69.4) |  | 1,063 (67.2) |  |
| Mild | 7 808 (24.0) | 133 (21.4) |  | 374 (23.6) |  |
| Moderate/severe | 2 861 (8.8) | 57 (9.2) |  | 145 (9.2) |  |
| **Nausea or vomiting** | **30,348 (84.0)** | **563 (66.2)** | **0.33** | **1,469 (82.2)** |  |
| No symptoms | 29,039 (95.7) | 534 (94.9) |  | 1,394 (94.9) | **0.15** |
| Any symptoms | 1,309 (4.3) | 29 (5.2) |  | 75 (5.1) |  |
| **Faintness or dizziness** | **30,535 (84.5)** | **575 (67.6)** | **0.28** | **1,485 (83.1)** |  |
| No symptoms | 27,948 (91.5) | 519 (90.3) |  | 1,317 (88.7) |  |
| Any symptoms | 2,587 (8.5) | 56 (9.7) |  | 168 (11.3) |  |
| **Wind** | **33,729 (93.4)** | **652 (76.7)** | **0.04** | **1,655 (92.6)** | **0.04** |
| None | 12,812 (38.0) | 278 (42.6) |  | 621 (37.5) |  |
| Mild | 14,161 (42.0) | 245 (37.6) |  | 661 (39.9) |  |
| Moderate/severe | 6,756 (20.0) | 129 (19.8) |  | 373 (22.5) |  |
| **Bottom soreness** | **31,532 (87.3)** | **599 (70.5)** | **0.28** | **1,547 (86.5)** | **0.004** |
| None | 22,265 (70.6) | 406 (67.8) |  | 1,038 (67.1) |  |
| Mild | 7,344 (23.3) | 156 (26.0) |  | 390 (25.2) |  |
| Moderate/severe | 1,923 (6.1) | 37 (6.2) |  | 119 (7.7) |  |
| **Soiling** | **30,531 (84.5)** | **576 (67.8)** | **<0.001** | **1,486 (83.1)** | **0.11** |
| No symptoms | 27,215 (89.1) | 486 (84.4) |  | 1 305 (87.8) |  |
| Any symptoms | 3,316 (10.9) | 90 (15.6) |  | 181 (12.2) |  |
| **Sleep disturbance** | **30,573 (84.6)** | **573 (67.4)** | **0.27** | **1,495 (83.6)** | **<0.001** |
| No symptoms | 27,683 (90.5) | 511 (89.2) |  | 1,302 (87.1) |  |
| Any symptoms | 2,890 (9.5) | 62 (10.8) |  | 193 (12.9) |  |

Abbreviations: FS=flexible sigmoidoscopy.

^a^ Participants who were referred for a same day repeat FS, barium enema or surgery were excluded. Non-attendance at referred procedure was only analysed in those referred for later day FS or colonoscopy and thus only they were included in the table and compared to non-referred participants.

^b^All n and percentage except the entry for age, which is median and interquartile range.

^c^P-value from X^2^ test of comparison between participants who were referred for a later day repeat FS and participants who were not referred for a repeat FS examination, colonoscopy, a barium enema or surgery.

^d^A total of eight participants were excluded from the colonoscopy analysis, which includes six participants who were too ill for a colonoscopy examination, one participant who had died before the examination and one participant who had moved away.

^e^P-value from X^2^ test of comparison between those participants who were referred for a colonoscopy and participants who were not referred for a repeat FS examination, colonoscopy, a barium enema or surgery.

**Supplementary Table 9.** Whether first flexible sigmoidoscopy examination was classed as technically inadequate by age, sex and test pain.

|  | **n (%)^a^** | **Technically inadequate first flexible sigmoidoscopy examination**  **n (%)^a,b^** | **Univariable**  **OR (95%CI)** | **p-value^c^** | **Multivariable**  **OR (95%CI)^d^** | **p-value^c^** |
| --- | --- | --- | --- | --- | --- | --- |
| **Total^e^** | **39,952 (100)** | **6,185 (15.5)** |  |  |  |  |
| **Age, years** | **60.4 (55.1-67.0)** | **60.7 (55.1-66.7)** | 1.03 (1.02-1.04) | <0.001 | - | - |
| **Sex** | **39,952 (100)** | **6,185 (15.5)** |  | <0.001 |  | - |
| Male | 20,109 (50.3) | 2,258 (11.2) | 1 |  | - |  |
| Female | 19,843 (49.7) | 3,927 (19.8) | 1.95 (1.84-2.06) |  | - |  |
| **Test pain^f^** | **38,807 (97.1)** | **5,857 (15.1)** |  | <0.001 |  | <0.001 |
| None | 10,566 (27.2) | 1,110 (10.5) | 1 |  | 1 |  |
| Mild | 20,452 (52.7) | 2,806 (13.7) | 1.35 (1.26-1.46) |  | 1.27 (1.18-1.37) |  |
| Quite a lot | 6,742 (17.4) | 1,565 (23.2) | 2.58 (2.37-2.80) |  | 2.27 (2.08-2.47) |  |
| Severe | 1,047 (2.7) | 376 (35.9) | 4.77 (4.15-5.50) |  | 4.04 (3.50-4.66) |  |

Abbreviations: CI=confidence interval. OR=odds ratio.

^a^All n and percentage except the entry for age, which is median and interquartile range.

^b^An inadequate examination was one satisfying any of the following: classed as incomplete, classed as unknown completeness but reached only the rectum, rectosigmoid or sigmoid colon, or had poor bowel preparation; while an adequate examination was one without poor bowel preparation that was either classed as complete or classed as unknown completeness and reached at least the sigmoid descending junction.

^c^Calculated with the likelihood ratio test.

^d^Multivariable model includes age and sex.

^e^189 missing values on technically inadequate exam variable.

^f^As reported by participants on the post first flexible sigmoidoscopy questionnaire. 1,145 missing values on test pain variable.

**Supplementary Table 10.** Total procedure time of first flexible sigmoidoscopy examination by post first flexible sigmoidoscopy questionnaire responses.

|  | **Total procedure time of first flexible sigmoidoscopy** | | | | |  |
| --- | --- | --- | --- | --- | --- | --- |
|  | **<3.00**  **minutes**  **n (%)** | **3.00-5.59**  **minutes**  **n (%)** | **6.00-8.59 minutes**  **n (%)** | **9.00-11.59 minutes**  **n (%)** | $\geq$**12.00**  **minutes**  **n (%)** | **p-value**^a^ |
| **Total^b^** | 7,114 (17.9) | 19,492 (48.9) | 7,807 (19.6) | 2,876 (7.2) | 2,563 (6.4) |  |
| **Test pain^c^** |  |  |  |  |  | < 0.001 |
| None | 2,210 (32.1) | 5,370 (28.3) | 1,812 (23.9) | 621 (22.2) | 527 (21.1) |  |
| Mild | 3,530 (51.4) | 10,011 (52.7) | 3,980 (52.5) | 1,535 (54.8) | 1,366 (54.6) |  |
| Quite a lot/severe | 1,131 (16.5) | 3,608 (19.0) | 1,795 (23.7) | 644 (23.0) | 610 (24.4) |  |
| **Expected pain^d^** |  |  |  |  |  | <0.001 |
| Less painful than expected | 3,262 (48.0) | 8,296 (44.1) | 2,880 (38.2) | 1,034 (37.2) | 910 (36.6) |  |
| About the same as expected | 2,421 (35.6) | 7,014 (37.3) | 2,959 (39.2) | 1,114 (40.0) | 964 (38.8) |  |
| More painful than expected | 1,118 (16.4) | 3,513 (18.7) | 1,707 (22.6) | 634 (22.8) | 612 (24.6) |  |
| **Abdominal pain/cramps^e^** |  |  |  |  |  | <0.001 |
| No symptoms | 4,414 (69.9) | 12,011 (68.5) | 5,524 (31.5) | 1,626 (62.9) | 1,409 (60.8) |  |
| Any symptoms | 1,900 (30.1) | 5,524 (31.5) | 2,481 (35.3) | 958 (37.1) | 909 (39.2) |  |
| **Nausea or vomiting^f^** |  |  |  |  |  | 0.530 |
| No symptoms | 5,658 (95.7) | 15,638 (95.7) | 6,192 (95.2) | 2,297 (95.6) | 2,045 (95.6) |  |
| Any symptoms | 254 (4.3) | 696 (4.3) | 311 (4.8) | 106 (4.4) | 95 (4.4) |  |
| **Faintness or dizziness^g^** |  |  |  |  |  | 0.004 |
| No symptoms | 5,452 (91.6) | 15,092 (91.6) | 5,924 (90.6) | 2,197 (90.7) | 1,920 (89.6) |  |
| Any symptoms | 497 (8.4) | 1,377 (8.4) | 613 (9.4) | 225 (9.3) | 222 (10.4) |  |
| **Wind^h^** |  |  |  |  |  | <0.001 |
| None | 2,670 (40.8) | 7,150 (39.4) | 2,608 (35.8) | 890 (33.2) | 746 (31.3) |  |
| Mild | 2,692 (41.1) | 7,581 (41.8) | 3,041 (41.7) | 1,132 (42.3) | 1,064 (44.6) |  |
| Moderate/severe | 1,186 (18.1) | 3,424 (18.9) | 1,640 (22.5) | 655 (24.5) | 577 (24.2) |  |
| **Bottom soreness^i^** |  |  |  |  |  | <0.001 |
| No symptoms | 4,239 (68.8) | 12,082 (71.2) | 4,684 (69.1) | 1,694 (67.3) | 1,480 (66.4) |  |
| Any symptoms | 1,920 (31.2) | 4,888 (28.8) | 2,092 (30.9) | 824 (32.7) | 750 (33.6) |  |
| **Soiling^j^** |  |  |  |  |  | <0.001 |
| No symptoms | 5,311 (89.2) | 14,696 (89.5) | 5,751 (87.8) | 2,109 (87.0) | 1,894 (87.5) |  |
| Any symptoms | 644 (10.8) | 1,729 (10.5) | 800 (12.2) | 314 (13.0) | 270 (12.5) |  |
| **Sleep disturbance^k^** |  |  |  |  |  | 0.003 |
| No symptoms | 5,401 (90.8) | 14,942 (90.6) | 5,880 (89.8) | 2,170 (90.1) | 1,896 (88.3) |  |
| Any symptoms | 547 (9.2) | 1,546 (9.4) | 671 (10.2) | 238 (9.9) | 252 (11.7) |  |

^a^Calculated using the X^2^ test.

^b^289 missing values on total procedure time (n=39,852).

^c^Test pain variable (n=38,750).

^d^Expected pain variable (n=38,438).

^e^Abdominal pain or cramps variable (n=35,770).

^f^Nausea or vomiting variable (n=33,292).

^g^Faintness or dizziness (n=33,519).

^h^Wind variable (n=37,056).

^i^Bottom soreness variable (n=34,653).

^j^Soiling variable (n=33,518).

^k^Sleep disturbance variable (n=33,543).

**Figure 1.** Cumulative distal colorectal cancer incidence by time from first FS examination among all participants (n=40,141).

**A.**

**B.**

**C.**

**D.**

**E.**

**F.**

**G.**

**H.**

**I.**

**J.**

**K.**

**L.**

**M.**
